# Halogen Bonding Heteroditopic Materials for Cooperative Sodium Iodide Binding and Extraction

**DOI:** 10.1002/chem.202102952

**Published:** 2021-10-01

**Authors:** Andrew Docker, James G. Stevens, Paul D. Beer

**Affiliations:** ^1^ Department of Chemistry University of Oxford Chemistry Research Laboratory Mansfield Road Oxford OX1 3TA; ^2^ Johnson Matthey Blount's Court Sonning Common, Reading RG4 9NH UK

**Keywords:** Halogen bonding, Ion-pair recognition, Sigma-hole interactions, Solid-liquid extraction

## Abstract

A series of novel heteroditopic halogen bonding (XB) receptor functionalised silica based materials, containing mono‐ and bis‐iodotriazole benzo‐15‐crown‐5 groups are investigated for the cooperative binding and extraction of sodium halide ion‐pair species from aqueous solution. Characterisation of the XB materials by CHN elemental analysis, ^13^C CP/MAS NMR and ATR‐FTIR spectroscopies confirms and quantifies the successful incorporation of the ion‐pair receptor frameworks to the silica material. ICP‐MS solid‐liquid extraction studies demonstrate the bidentate XB functionalised material is capable of NaI extraction from water. Importantly, cooperative XB‐mediated sodium halide ion‐pair binding is determined to be crucial to the material's extraction capabilities, impressively demonstrating a two‐fold enhancement in sodium iodide extraction efficiency relative to a heteroditopic hydrogen bonding receptor functionalised silica material analogue.

## Graphical abstract

**A series of halogen bonding (XB) heteroditopic ion‐pair receptors** comprising of benzo15‐crown‐5 cation binding moieties and iodotriazole XB donors are covalently integrated into silica QuadraSil^TM^ based solid supports. Investigations into their sodium halide extraction capabilities demonstrated their ability as NaI extraction agents from water. Comparison with HB analogues demonstrate the crucial role of XB‐mediated NaI ion‐pair binding in the extraction performance.

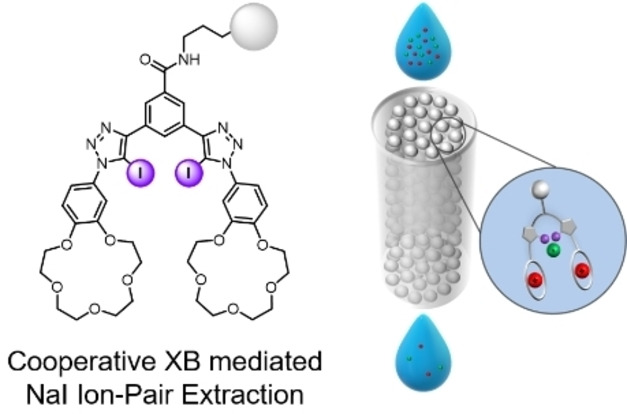

Heteroditopic receptors, possessing binding sites for both cations and anions, frequently exhibit augmented recognition behaviour relative to their monotopic counterparts.[^1^, ^2^, ^3^, ^4^] The source of this modulated binding behaviour, or cooperativity, is generally attributed to favourable electrostatic interactions between the proximal co‐bound species and/or conformational effects upon ion‐pair binding. The rational employment of these effects in ion‐pair receptor design has been demonstrated to be a powerful strategy in fine‐tuning not only ion‐affinity, but also selectivity profiles.[^5^, ^6^, ^7^, ^8^, ^9^, ^10^, ^11^, ^12^] Advantageously, these effects frequently translate to highly desirable functionalities leading to their successful application in fields such as sensing,[^13^, ^14^, ^15^] molecular logic gates[^16^, ^17^] and membrane transport.[^18^, ^19^, ^20^] In particular, the application of heteroditopic molecular receptor systems as novel extraction agents has demonstrated enormous promise as an alternative supramolecular strategy to sequester and/or recover a range of alkali‐, transition‐ and precious‐metal salts.[^5^, ^21^, ^22^, ^23^, ^24^] Sessler and others have elegantly shown that the incorporation of ion‐pair receptor frameworks into polymeric or resin‐based solid supports serves to not only provide a reusable extractant material, but often through subtle multivalent effects enhances extraction efficiencies relative to a discrete molecular receptor analogue.[^25^, ^26^, ^27^, ^28^] Notwithstanding the enormous progress that has been made in heteroditopic receptor design over the last few decades, the means by which anion binding is achieved is overwhelmingly dominated by hydrogen bonding systems (HB). However, the recent emergence of anion recognition strategies employing sigma‐hole based interactions such as halogen bonding (XB) and chalcogen bonding (ChB) has notably signified these non‐covalent interactions to engender unique advantages associated with their use.[^29^, ^30^, ^31^, ^32^, ^33^, ^34^, ^35^, ^36^, ^37^, ^38^, ^39^, ^40^, ^41^] Indeed, rarer still is the incorporation of sigma‐hole donors to serve as the anion recognition component in heteroditopic receptor systems.[^42^, ^43^, ^44^, ^45^, ^46^] We have recently reported a 3,5‐bis‐iodotriazole pyridine XB receptor functionalised with pendant benzo‐15‐crown‐5 (B15 C5) moieties displayed a remarkable ‘switch‐on’ of anion binding potency, in the presence of co‐bound sodium cations, in which the source of this enhancement was determined to be a cation induced through‐bond polarisation of the sigma‐hole XB donor.^[47]^ Capitalising on this ion‐pair recognition behaviour, we sought to exploit this unique cooperativity mechanism to develop functional solid‐immobilised XB‐mediated extraction materials. Herein, we describe a series of XB and HB heteroditopic receptors covalently linked to a silica‐based QuadraSil^TM^ solid support material (Figure 1). Importantly, ICP‐MS monitored extraction studies reveal the potency and unique cooperativity mechanism of XB mediated ion‐pair recognition is crucial to the material's extraction performance of NaI from water.

**Figure 1:**
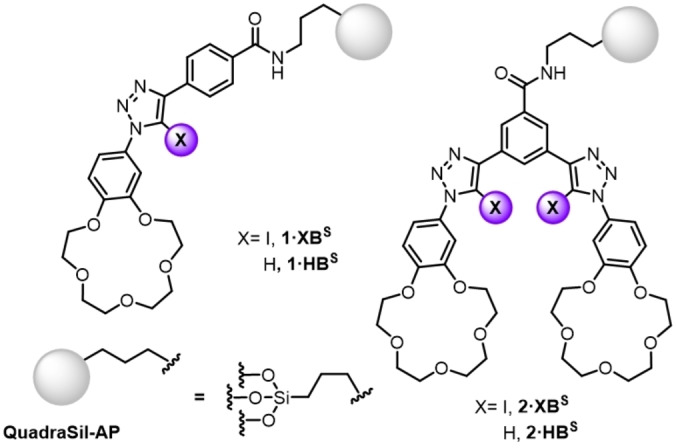
Target XB and HB heteroditopic receptor functionalised extraction materials.

## Results and Discussion

### Design and synthesis of XB and HB heteroditopic receptor functionalised silica materials

The synthetic strategy undertaken for the preparation of the target XB and HB heteroditopic receptor functionalised silica materials involved the initial synthesis of mono‐ and bidentate iodotriazole phenyl motifs functionalised with B15 C5 groups, wherein the appendage of a ethylparaben ester moiety would allow solid silica‐based support immobilisation via amide condensation with amino‐propyl groups of QuadraSil^TM^‐AP.

**Scheme 1:**
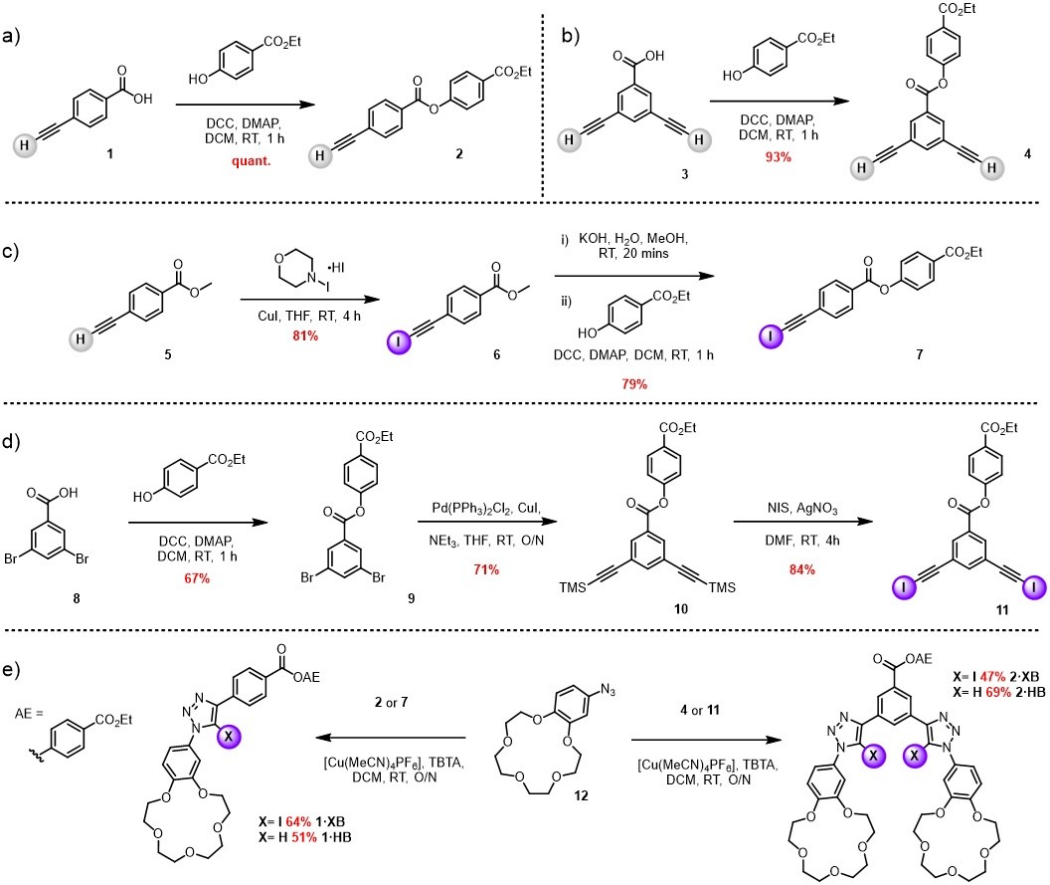
a), b), c) and d) Synthesis of appropriately functionalised mono‐ and bis‐alkyne precursors. e) CuAAC synthesis of the ethylparaben activated ester appended XB and HB heteroditopic receptors.

Accordingly, the target XB and HB receptors, were synthesised via a copper‐catalysed azide alkyne cycloaddition (CuAAC) reaction between the appropriately functionalised alkyne precursors and azido‐benzo‐15‐crown‐5. The requisite mono‐ and bis‐ alkyne precursors were synthesized according to Scheme 1. In the case of the target proto‐alkynes, a DCC mediated coupling reaction between the parent acids, **1** and **3**, and 4‐hydroxyethylbenzoate (ethylparaben) in the presence of a catalytic amount of DMAP afforded the esters **2** and **4** good yields. Iodo‐alkyne **7** was synthesized in a similar manner, in which an iodination reaction of methyl ester **5** with *N*‐iodomorpholine hydroiodide/CuI, afforded **6**, in 81 % yield. Subsequently the iodo‐alkyne **6** was subjected to carefully controlled basic hydrolysis and esterified with ethylparaben under similar coupling conditions and isolated by column chromatography to afford **7** in 79 % yield over three steps. The analogous bis‐iodoalkyne, was synthesised via a standard Sonogashira coupling reaction with two equivalents of TMS‐acetylene and ester **9** to afford the bis‐TMS protected alkyne **10** in 71 % yield. Simultaneous TMS‐deprotection and iodination of the bis‐alkyne was achieved via treatment with N‐iodosuccinimide in DMF in the presence of catalytic AgNO_3_ to afford target bis‐iodoalkyne **11** in 84 % yield without the need for further purification.

The isolated ester‐appended mono‐ and bis‐ functionalised alkynes were subjected to CuAAC reactions, in the presence of catalytic [Cu(MeCN)_4_]PF_6_ and the rate accelerating ligand tris(benzyltriazolyl) amine (TBTA) with either 1.1 or 2.2 equivalents of B15 C5 azide **12**, respectively. In all cases, after ca. 12 h of stirring under an atmosphere of nitrogen at room temperature, TLC analysis of the crude reaction mixtures indicated complete consumption of the alkyne starting material and ESI‐MS confirmed the formation of the corresponding triazole products. The reaction mixtures were subjected to an NH_4_OH/EDTA_(aq)_ work up procedure after which the respective crude mixture was purified by iterative trituration protocols (see Supporting Information) using diethyl ether and methanol to afford the receptors **1 ⋅ XB**, **1 ⋅ HB**, **2 ⋅ XB** and **2 ⋅ HB** in yields in the range of 47–69 %. All of the heteroditopic receptors were characterised by ^1^H and ^13^C NMR and high resolution mass spectrometry.

With the series of activated ester‐appended heteroditopic receptors in hand, attention then turned to their immobilization on amino‐propyl functionalised QuadraSil^TM^‐AP (AP) solid support via an amide condensation reaction. In a general procedure, a dichloromethane solution of the receptor, containing five equivalents of the receptor relative to the average concentration of amino‐propyl functional groups for a given mass of QuadraSil^TM^‐AP_,_ was allowed to react at room temperature with a suspension of the silica‐support. After three days the suspensions were decanted and under vacuum filtration, copious washing of the functionalised silica materials with CH_2_Cl_2_/MeOH mixtures was undertaken (See Supporting Information for synthetic details), ensuring the removal of unreacted organic material and the ethylparaben aryl alcohol by‐product of the amide condensation, furnishing the family of mono‐ and bidentate XB and HB heteroditopic silica‐based materials **1 ⋅ XB^S^**, **1 ⋅ HB^S^**, **2 ⋅ XB^S^** and **2 ⋅ HB^S^**.

**Figure 2:**
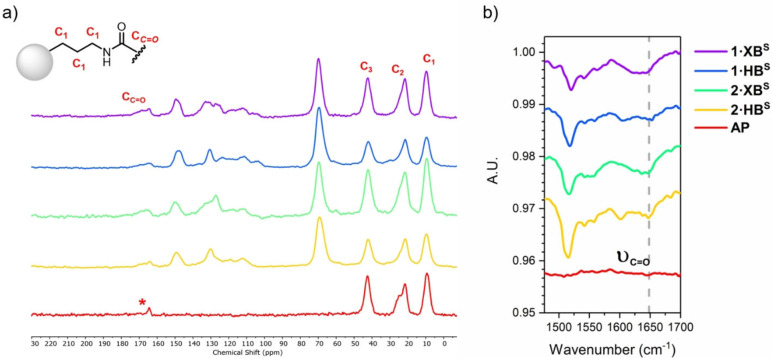
a) ^13^C CP/MAS‐NMR spectrum and b) truncated ATR‐FTIR spectrum of the extractant materials **1 ⋅ XB^S^**, **1 ⋅ HB^S^**, **2 ⋅ XB^S^** and **2 ⋅ HB^S^** and the parent **AP**. Asterisk indicates the resonance arising from trace carbamate formation due to presence of atmospheric CO_2_.

Characterisation of the resultant materials by solid state ^13^C CP/MAS NMR (Figure 2a) revealed that in addition to the three characteristic signals corresponding to the constituent methylene groups of the amino‐propyl (C_1_–C_3_), three new distinct regions are identifiable. The broad resonances in the range 60 and 80 ppm, are consistent with the introduction of additional aliphatic environments, attributed to the methylene benzo‐15‐crown‐5 units. A complex series of signals in the range 100–150 ppm are also observed, consistent with the introduction of multiple aromatic carbon environments deriving from the respective receptor scaffold. Further evidence for the successful covalent attachment of the heteroditopic receptors to the silica‐support material via the anticipated amide linkage was noted by the appearance of a characteristic amide carbon signal in the range 170–180 ppm, together with FTIR analysis of the materials revealing a new sharp absorption at approximately 1650 cm^−1^ corresponding to the carbonyl stretching frequency for an amide (Figure 2b).

Quantifying the amount of respective heteroditopic receptor incorporated into the sorbent material was achieved by CHN combustion analysis, which determined the loadings for the four materials to be in the range of 0.23–0.46 mmolg^−1^ (Table 1).

**Table 1:** CHN analysis determined receptor loadings and amino‐propyl functionalisation percentages.

Material	Loading/mmolg^−1 [a]^	Amino‐Propyl Functionalisation^[c]^
**1 ⋅ XB^S^**	0.357	30 %
**1 ⋅ HB^S^**	0.445	24 %
**2 ⋅ XB^S^**	0.229	15 %
**2 ⋅ HB^S^**	0.231	16 %
**AP**	1.49^[b]^	–

[a] Loading calculated on the basis CHN analysis and refers to the loading per receptor. [b] Refers to the determined loading of amino‐propyl groups and is consistent with manufacturers specification of 1.5–2.0 mmolg^−1^. [c] Refers to the calculated percentage of amino‐propyl groups that have undergone an amide coupling reaction.

Inspection of Table 1 reveals that CHN analysis calculation of amino‐propyl group loading of the parent AP material is 1.49 mmolg^−1^ and is consistent with the specification of 1.50–2.0 mmolg^−1^ as specified by the manufacturer. Importantly, this value facilitated the reliable determination of receptor coverage of the material. Surveying the percentage functionalisation of the isolated materials reveals that the silica surface condensation reaction with the mono‐substituted triazole‐B15 C5 derivatives was more efficient relative to the bidentate receptors. This is most likely attributable to the considerable steric demand of reactions occurring at solid interfaces.

### Preliminary extraction studies

The heteroditopic XB and HB receptor functionalised silica based materials were subjected to preliminary solid‐liquid extraction experiments, specifically the extraction of sodium halide ion‐pairs from aqueous solution. In a typical experiment a given quantity of the silica‐based extractant material was exposed to a fixed volume of a 5 mM NaX_(aq)_ solution (X=Cl^−^, Br^−^, I^−^ and NO_3_^−^). To allow for full equilibration of the extraction process, the mixture was subjected to agitation on a roller mixer for 16 h. After which time the respective solutions were centrifuged, filtered (using a 0.45 μm syringe filter) and the resulting solution subjected to ICP‐MS and ion chromatography analysis against known standards facilitating quantitative determination of anion concentration before and after exposure to the functionalised silica‐based materials. In all cases a depletion of X^−^ concentration was only observed when the materials were treated with sodium iodide solutions. This apparent Hofmeister bias correlates with the respective thermodynamic parameters of the sodium salts (e. g. respective anion hydration and lattice enthalpies). Table 2 summarises the calculated iodide extraction per μmol of the materials in which extraction efficiency relative to **1 ⋅ HB^S^** is also presented for comparison.

**Table 2:** Summarised ICP‐MS determined iodide extraction capabilities.

Material	Iodide extraction/ ppm μmol^−1 [a]^	Relative extraction efficiency^[b]^
**1 ⋅ XB^S^**	0.0557	1.3
**1 ⋅ HB^S^**	0.0445	1
**2 ⋅ XB^S^**	0.330	7.4
**2 ⋅ HB^S^**	0.317	4.9

[a] Calculated from the ICP‐MS measured depletion of iodide concentration in the post extraction solution for a given mass of extractant material used accounting for loading of heteroditopic receptor. [b] Iodide extraction capability relative to **1 ⋅ HB^S^**. Errors estimated to be 10 %.

In order to eliminate the possibility of non‐specific adsorption of ionic species to the silica materials, the parent unfunctionalised AP material was also subjected to the same procedures, however in all cases no depletion of counteranion was observed. Close inspection of the extraction performances reveals that the sodium iodide extraction capabilities of the bidentate heteroditopic receptor functionalised silica materials **2 ⋅ XB^S^** and **2 ⋅ HB^S^** are considerably enhanced (at least a four‐fold enhancement) relative to their monodentate material analogues **1 ⋅ XB^S^** and **1 ⋅ HB^S^** despite their reduced receptor loading values (Table 1). This increase in extraction efficiency between the mono‐ and bidentate systems can most likely be attributed to two main factors: (i) by multivalent effects arising from increased surface concentration of B15 C5 cation binding moieties and iodotriazole XB donors and (ii) a sodium cation induced polarisation of the C−I, in effect ‘switching on’ the XB donor anion binding potency. Notably the observed, almost, two‐fold increase in iodide extraction capability of **2 ⋅ XB^S^** relative to **2 ⋅ HB^S^** serves to highlight the superiority of XB over HB for cooperative sodium iodide extraction with bidentate heteroditopic receptor functionalisation.

## Conclusions

A series of novel XB and HB heteroditopic receptors were synthesised, comprising of B15 C5 cation binding groups covalently linked to either an iodo/proto triazole XB/HB donor. The appendage of an ethylparaben active ester group enabled the facile immobilisation of mono‐ and bidentate heteroditopic receptor systems to a amino‐propyl functionalised QuadraSil^TM^ support via amide bond formation. Characterisation of the four extractant materials by ^13^C CP/MAS NMR NMR and FTIR spectroscopy confirmed the successful covalent functionalisation of the silica based support. Furthermore, CHN elemental analysis of the isolated materials facilitated quantification of receptor loadings. Preliminary solid‐liquid ICP‐MS extraction studies with a range of sodium salts (NaX, X=Cl^−^, Br^−^, I^−^ and NO_3_^−^) revealed exclusive extraction of sodium iodide, where the bidentate receptor functionalised materials **2 ⋅ XB^S^** and **2 ⋅ HB^S^** displayed significantly enhanced sodium iodide extraction capabilities relative to their monodentate analogues by at least a factor of 4. Crucially, the determined NaI extraction capability of the XB‐based receptor **2 ⋅ XB^S^**, is enhanced two‐fold relative to the HB analogue **2 ⋅ HB^S^**, which is attributed to a combination of enhanced XB‐mediated NaI affinity and the unique through‐bond co‐bound cation induced polarisation mechanism observed in sigma‐hole based heteroditopic receptor systems. These results not only serve to illustrate the advantages conferred by exploiting the unique nature of sigma‐hole recognition behaviours, but in the context of functional materials demonstrates an exciting opportunity for the real‐world application of sigma‐hole mediated recognition in the context of salt recovery.

## Conflict of interest

The authors declare no conflict of interest.

## Supporting information

As a service to our authors and readers, this journal provides supporting information supplied by the authors. Such materials are peer reviewed and may be re‐organized for online delivery, but are not copy‐edited or typeset. Technical support issues arising from supporting information (other than missing files) should be addressed to the authors.

Supporting Information
