# Supplementary material for: Halogen Bonding Heteroditopic Materials for Cooperative Sodium Iodide Binding and Extraction
Source: Chemistry. 2021 Oct 1;27(59):14600–4. doi: 10.1002/chem.202102952 (PMC8596695; doi:10.1002/chem.202102952)
Supplement: Supplementary file 1 — Supporting Information [file CHEM-27-14600-s001.pdf]

Chemistry–A European Journal

Supporting Information

Halogen Bonding Heteroditopic Materials for Cooperative Sodium Iodide Binding and Extraction

Andrew Docker, James G. Stevens, and Paul D. Beer*

Materials and methods

All solvents and reagents were purchased from commercial suppliers and used as received unless otherwise stated. Dry solvents were obtained by purging with nitrogen and then passing through an MBraun MPSP-800 column. H_2O was de-ionized and micro filtered using a Milli-Q ® Millipore machine. Column chromatography was carried out on Merck® silica gel 60 under a positive pressure of nitrogen. Routine NMR spectra were recorded on either a Varian Mercury 300, a Bruker AVIII 400 or a Bruker AVIII 500 spectrometer with ^{1}H NMR titrations recorded on a Bruker AVIII 500 spectrometer. Solid state 400MHz is a Bruker Avance III HD Solid state NMR equipped with a 9.4T magnet. (^{13}C 100.6 MHz). Chemical shifts are quoted in parts per million relative to the residual solvent peak. Mass spectra were recorded on a Bruker µTOF spectrometer. Triethylamine was distilled from and stored over potassium hydroxide. Tris[(1-benzyl-1H-1,2,3-triazol-4-yl)methyl]amine (TBTA). The following compounds were prepared according to literature procedures **1**,[1] **3**,[2] **5**,[3] and **12**.[4]

General Procedure 1

The appropriate acid was suspended in anhydrous CH_2Cl_2 (ca. 20 ml per 0.5 g of acid) to which was added DCC (1.1 equivalents), ethyl 4-hydroxybenzoate (1.2 equivalents) and a catalytic amount of DMAP. The reaction mixture was left to stir at room temperature, until consumption of the acid was determined by TLC analysis (ca. 3 hours). After which time the solution was filtered and the solid carefully washed with CH_2Cl_2 (ca. 20 ml), the filtrate was collected and concentrated in vacuo. The resultant residue was subjected to silica gel column chromatography to afford the desired ester product.

General Procedure 2

$[Cu(MeCN)_4]PF_6$ (0.1 equivalents per alkyne) and TBTA (0.1 equivalents per alkyne) were dissolved in the minimum amount anhydrous degassed CH_2Cl_2 (ca. 5 ml) and left to stir for 15 minutes. After which time the appropriate alkyne was added to the solution as a solid, followed by azido-benzo-15-crown-5 (1.1 equivalents per alkyne). Once complete, as determined by TLC analysis, the reaction mixture was diluted with CH_2Cl_2 (ca. 150 ml) and washed with EDTA/$NH_4OH_{(aq)}$ solution (20 ml) and water (50 ml), the collected organic phase was dried over $MgSO_4$ and concentrated to dryness. The crude solid obtained was washed with Et_2O (10 × 10 ml) followed by MeOH (10 × 10 ml) followed by Et_2O (10 × 10 ml) to obtain the desired CuAAC product as an off white solid.

General Procedure 3

A CH_2Cl_2 solution of the appropriate receptor (5 equivalents per amino-propyl for a given mass QuadraSil-AP used) was exposed to QuadraSil-AP and stirred at room temperature for 3 days. After which the mixture was filtered, and the solid material was washed with CH_2Cl_2 (ca. 500 ml).

General Extraction Studies Procedure

A typical extraction procedure was conducted as follows; a known amount of a given silica-based material (ca. 20 mg) is exposed to a (1 ml) centrifuge tube to which was added a 5 mM solution of a NaX (X= Cl^-, Br^-, I^- and NO_3^-) aqueous solution. The sealed centrifuge tube was placed on a roller mixer and left mixing for 12 hours to allow equilibration. After this time the tube was placed in a centrifuge and the solution pipetted off, diluted to the required concentration, and subjected to ICP-MS analysis.

2, **4**, **9** were synthesised according to general procedure 1.

1·HB, **1·XB**, **2·HB** and **2·XB** were synthesised according to general procedure 2.

1·HB^S, **1·XB^S**, **2·HB^S** and **2·XB^S** were synthesised according to general procedure 3.

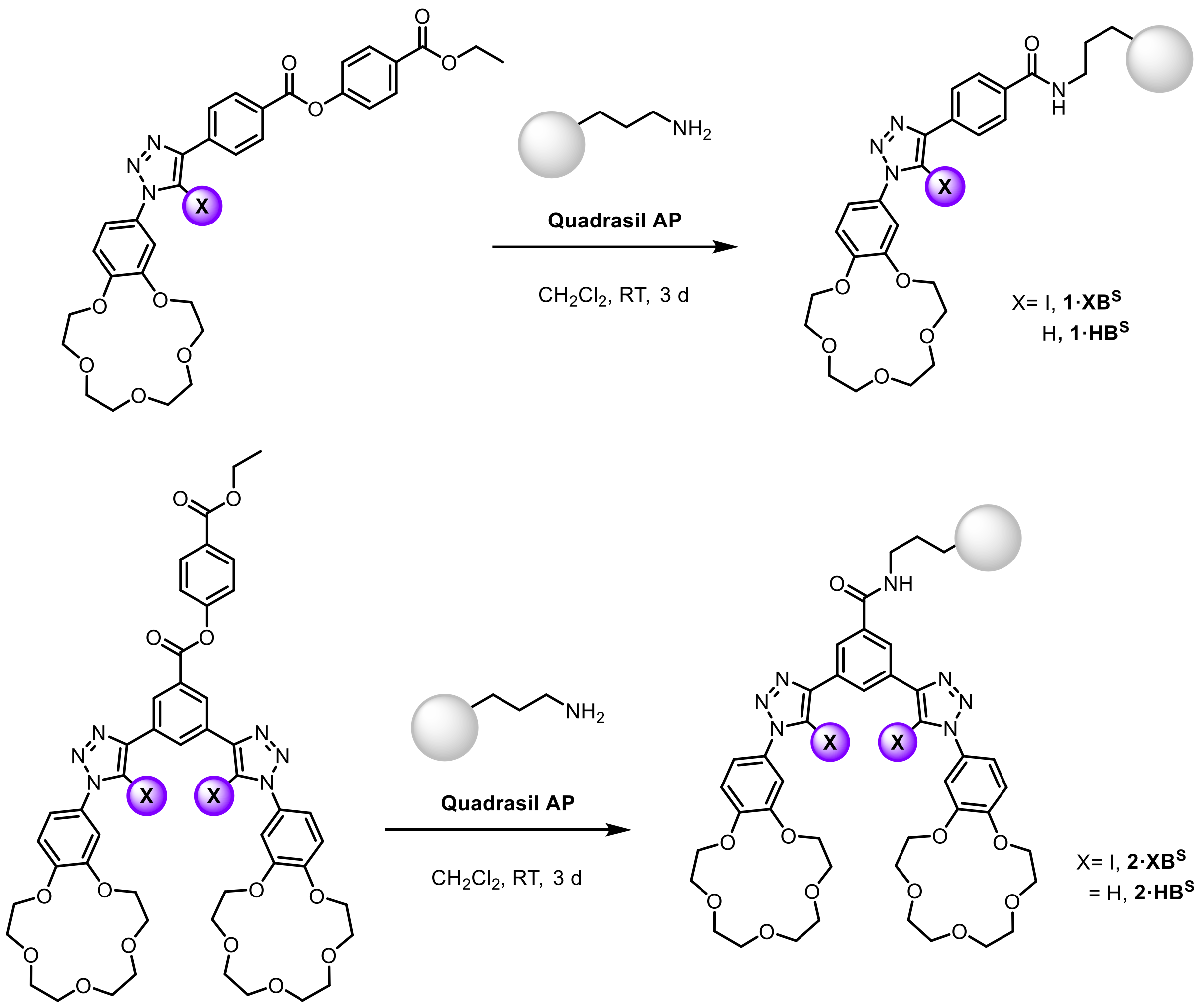

Scheme S1. Synthesis of the heteroditopic receptor functionalised QuadraSil-AP materials **1·XB**^{S}, **1·HB**^{S}, **2·XB**^{S} and **2·HB**^{S}.

2

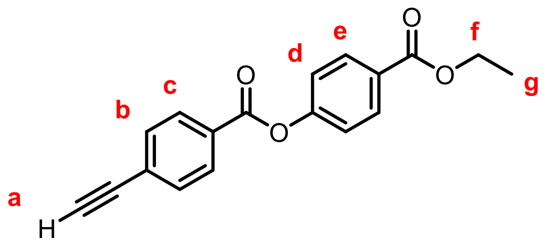

^{1}H NMR (400 MHz, $CDCl_3$) δ 8.14 (m, $4H_{b,c}$), 7.69 – 7.56 (m, $2H_e$), 7.35 – 7.27 (m, $2H_d$), 4.39 (q, *J* = 7.2 Hz, $2H_f$), 3.29 (s, $1H_a$), 1.40 (t, *J* = 7.2 Hz, $3H_g$).

^{13}C NMR (101 MHz, $CDCl_3$) δ 165.94, 164.11, 154.48, 132.46, 131.34, 130.23, 129.19, 128.41, 127.90, 121.75, 82.73, 80.93, 61.26, 14.47.

HRMS (ESI+ve) m/z: 295.09644 ($[M+H]^+$, $C_{18}H_{15}O_4$ requires 295.09649).

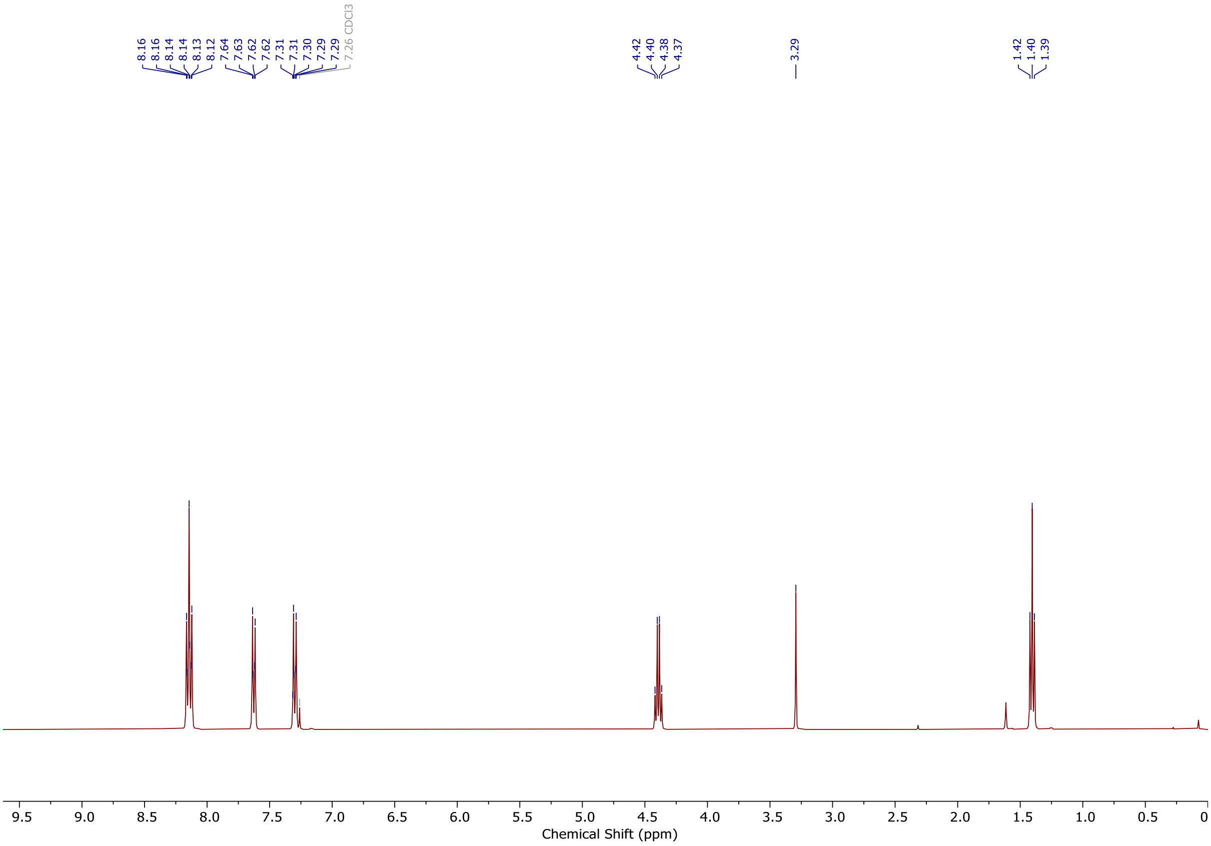

Figure S1. ^{1}H NMR Spectrum of **2** ($CDCl_3$, 400 MHz, 298K).

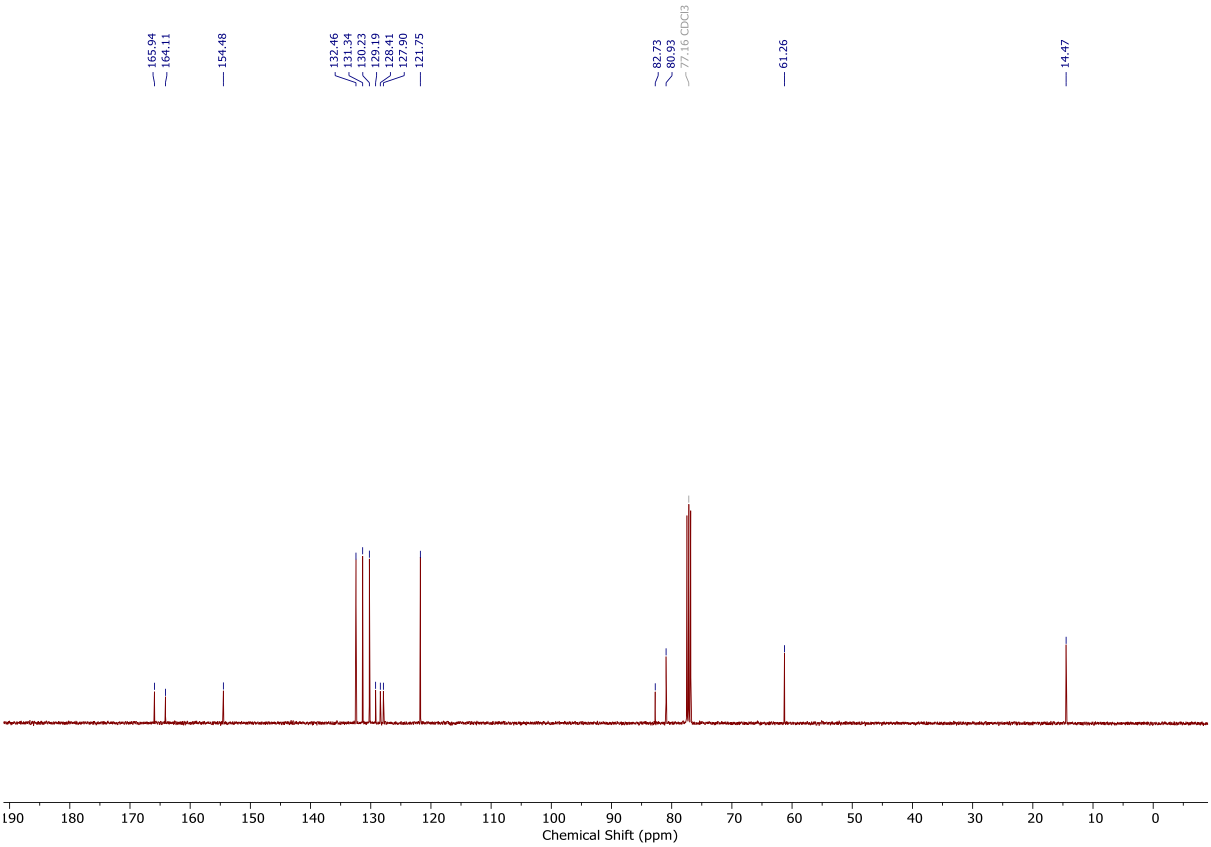

Figure S2. ^{13}C NMR Spectrum of **2** ($CDCl_3$, 101 MHz, 298K).

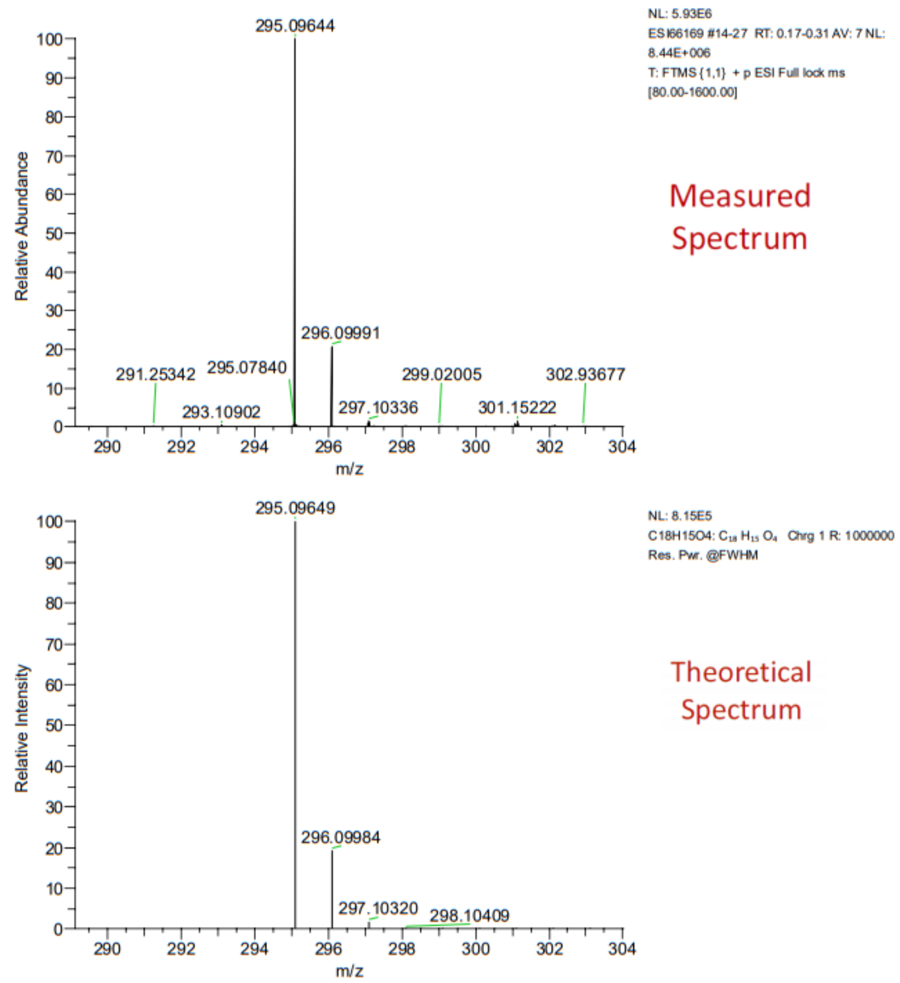

Figure S3. HRESI spectrum of **2**.

7

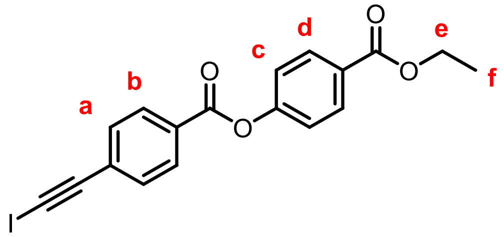

5 (250 mg, 1.56 mmol), N-iodomorpholine hydroiodide (798 mg, 2.34 mmol) and CuI (30 mg, 0.156 mmol) were dissolved in anhydrous THF (14 ml) and left to stir, excluded from light, for 4 hours at room temperature. After which time the mixture diluted with CH_2Cl_2 (ca. 100 ml) and filtered through an alumina plug. Silica was added to the resultant filtrate and concentrated to dryness. The silica-adsorbed compound was eluted with EtOAc:hexane (1:9, v/v) mixtures, and the collected filtrate concentrated to dryness to afford iodo-alkyne **6** and used immediately for the next step. **6** was dissolved in MeOH (15 ml), to which was added H_2O (1.5 ml), to this solution an aqueous solution of KOH (9 mg, 0.156 mmol), dissolved in H_2O (1.5 ml), was added dropwise. The reaction mixture was left to stir at room temperature excluded from light, until determined complete by TLC analysis (ca. 1 hr). After which time the reaction mixture was diluted with 1 M $HCl_{(aq)}$ until determined to in the pH range 2-3, the resultant aqueous phase was extracted into EtOAc (3 × 50 ml). The combined organic phases were washed with H_2O (2 × 20 ml), and dried over $MgSO_4$ to afford the alkyne-acid intermediate. The solid isolated as a dark yellow solid was subsequently subjected to general procedure 1, excluded from light throughout, to afford **7** as white solid in 79% yield over three steps.

^{1}H NMR (500 MHz, $CDCl_3$) δ 8.23 – 8.07 (m, 4$H_{a,b}$), 7.62 – 7.48 (m, 2H_d), 7.35 – 7.27 (m, 2H_c), 4.39 (q, *J* = 7.2 Hz, 2H_e), 1.41 (t, *J* = 7.2 Hz, 3H_f).

^{13}C NMR (126 MHz, $CDCl_3$) δ 165.96, 164.10, 154.50, 132.68, 131.36, 130.20, 129.08, 129.03, 128.42, 121.76, 93.45, 61.28, 14.48, 11.74.

HRMS (ESI+ve) m/z: 420.99292 ($[M+H]^+$, $C_{18}H_{14}O_4I$ requires 420.99313).

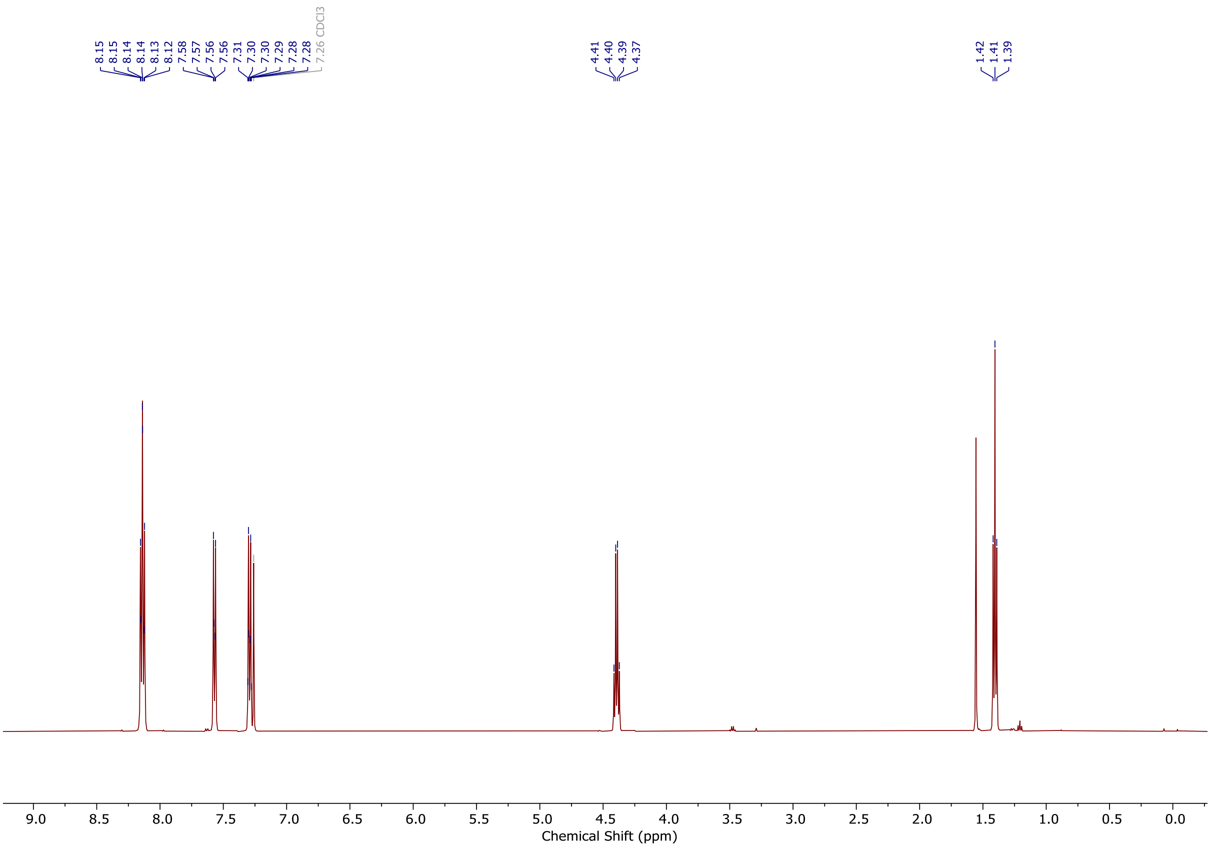

Figure S4. ^{1}H NMR Spectrum of **7** ($CDCl_3$, 500 MHz, 298K).

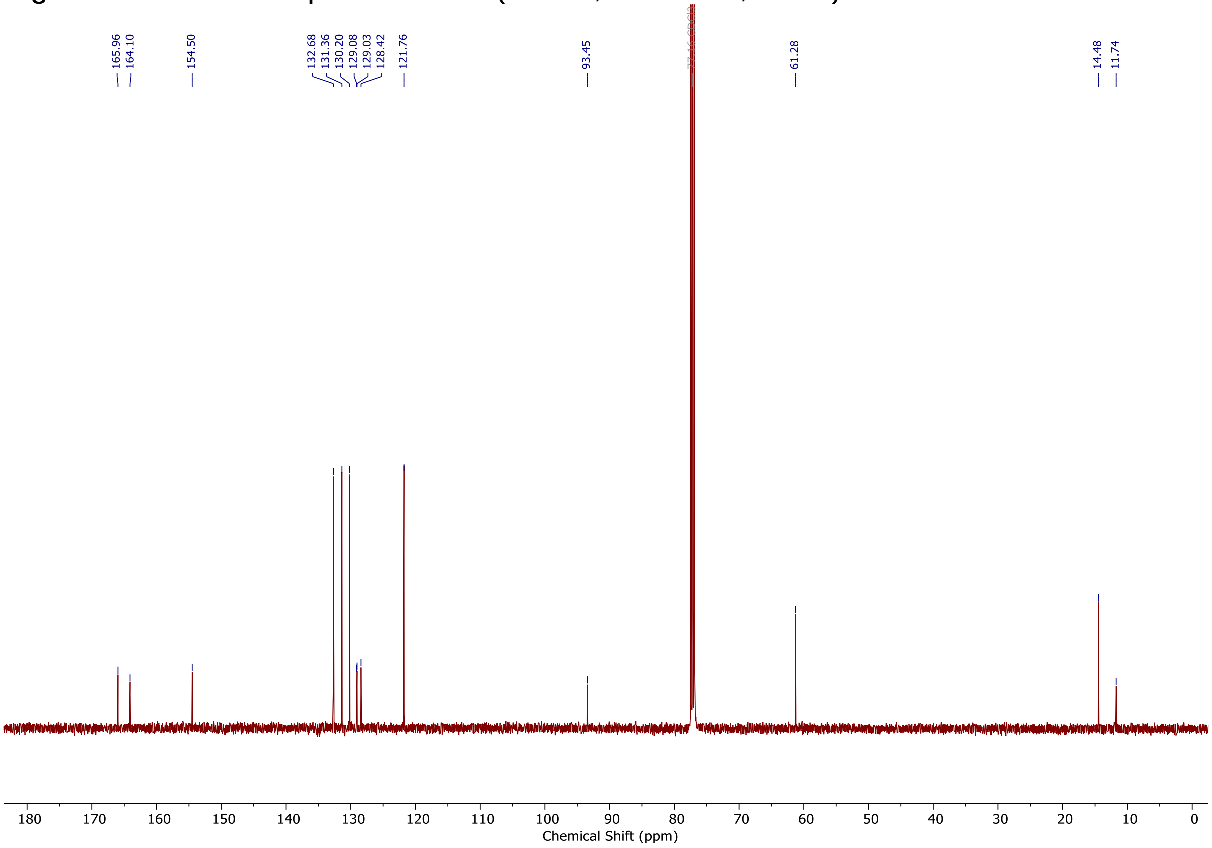

Figure S5. ^{13}C NMR Spectrum of **7** ($CDCl_3$, 126 MHz, 298K).

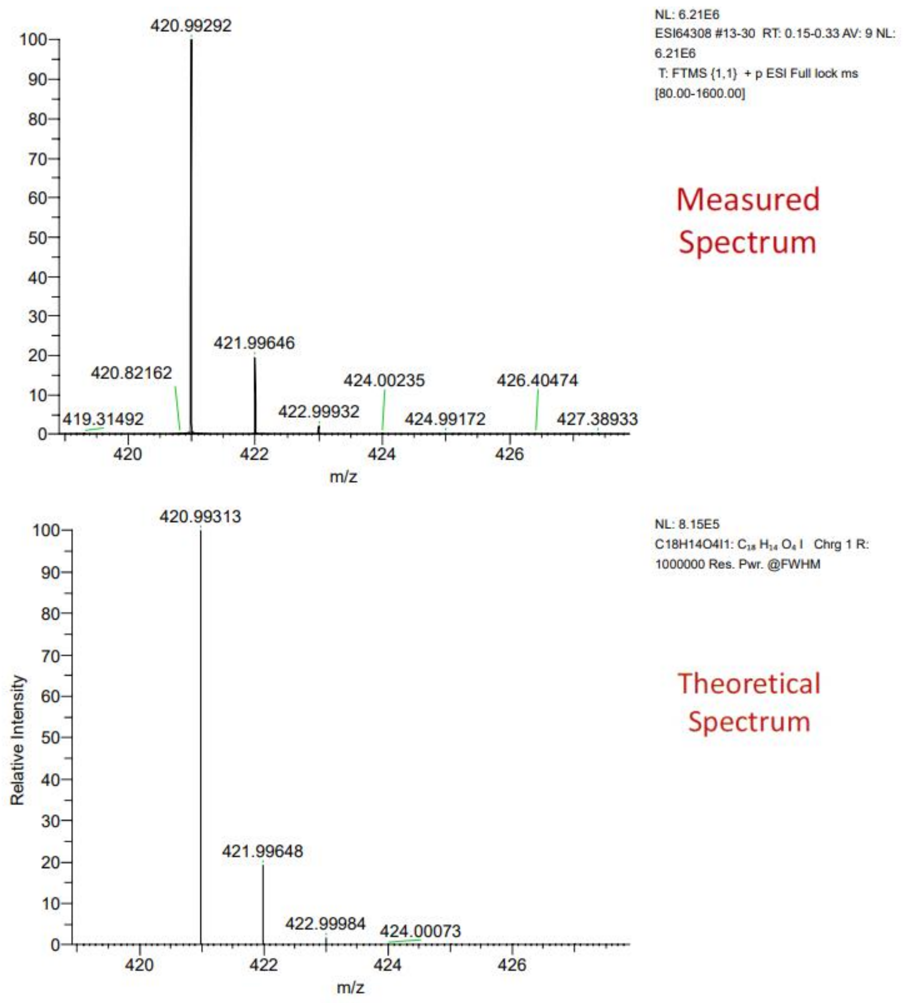

Figure S6. HRESI spectrum of **7**.

1·XB

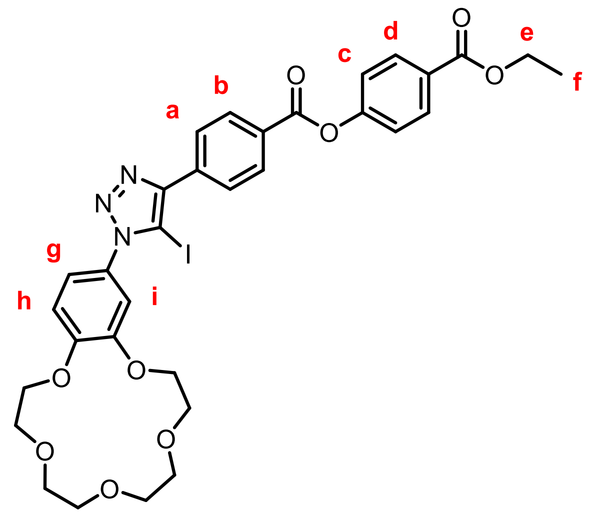

^{1}H NMR (500 MHz, $CDCl_3$) δ 8.33 (d, J = 8.4 Hz, $2H_b$), 8.23 (d, J = 8.4 Hz, $2H_a$), 8.19 – 8.12 (m, $2H_d$), 7.35 (m, $2H_c$), 7.12 – 6.95 (m, $3H_{g,h,i}$), 4.40 (q, J = 7.1 Hz, $2H_e$), 4.21 (m, 2H), 3.95 (m, 2H), 3.78 (q, J = 4.1, 3.5 Hz, 8H), 1.41 (t, J = 7.1 Hz, $3H_f$).

^{13}C NMR (126 MHz, $CDCl_3$) δ 165.92, 164.36, 154.57, 150.77, 149.41, 148.74, 135.61, 131.28, 130.62, 129.88, 129.01, 128.27, 127.65, 121.79, 119.61, 113.10, 112.23, 71.21, 70.43, 69.40, 69.32, 69.22, 69.13, 61.20, 14.43.

HRMS (ESI+ve) m/z: 730.12561 ($[M+H]^+$, $C_{32}H_{33}O_9N_3I$ requires 730.12560).

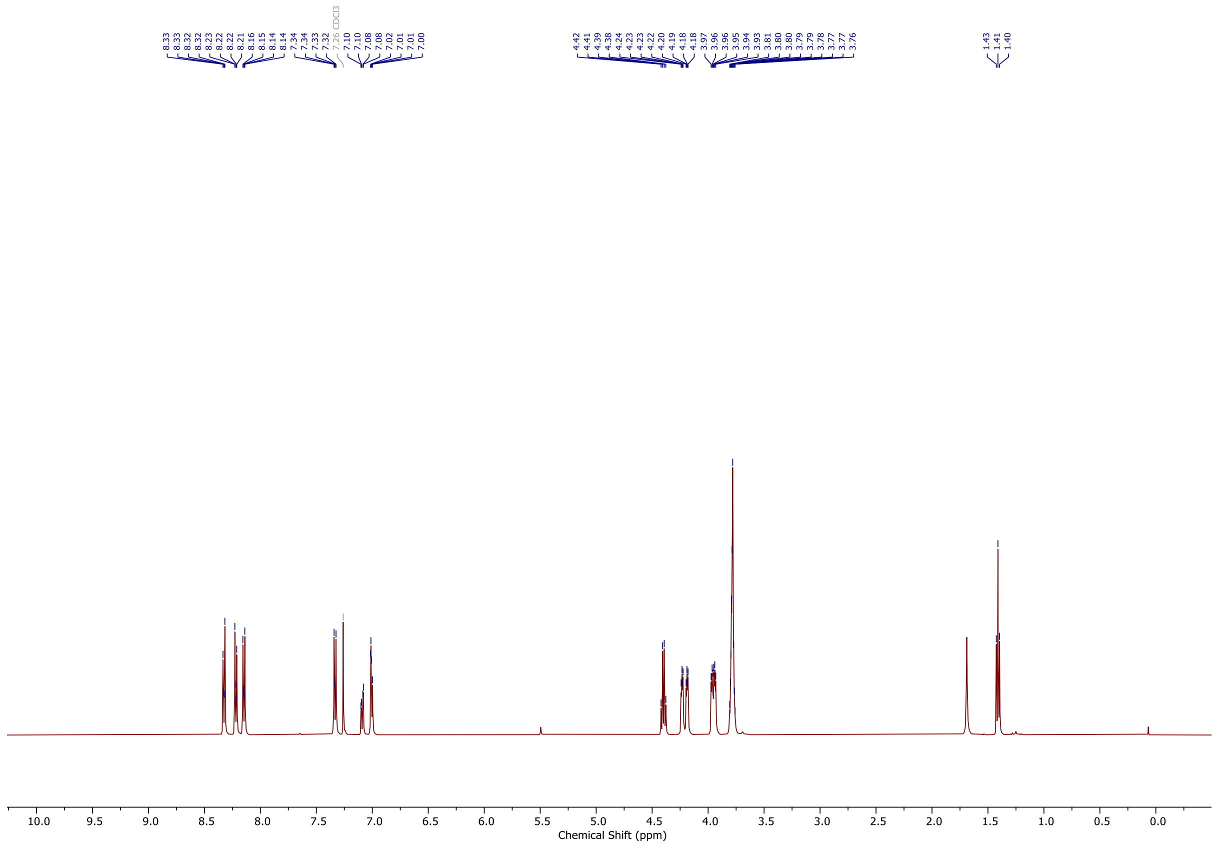

Figure S7. ^{1}H NMR Spectrum of **1·XB** ($CDCl_3$, 500 MHz, 298K).

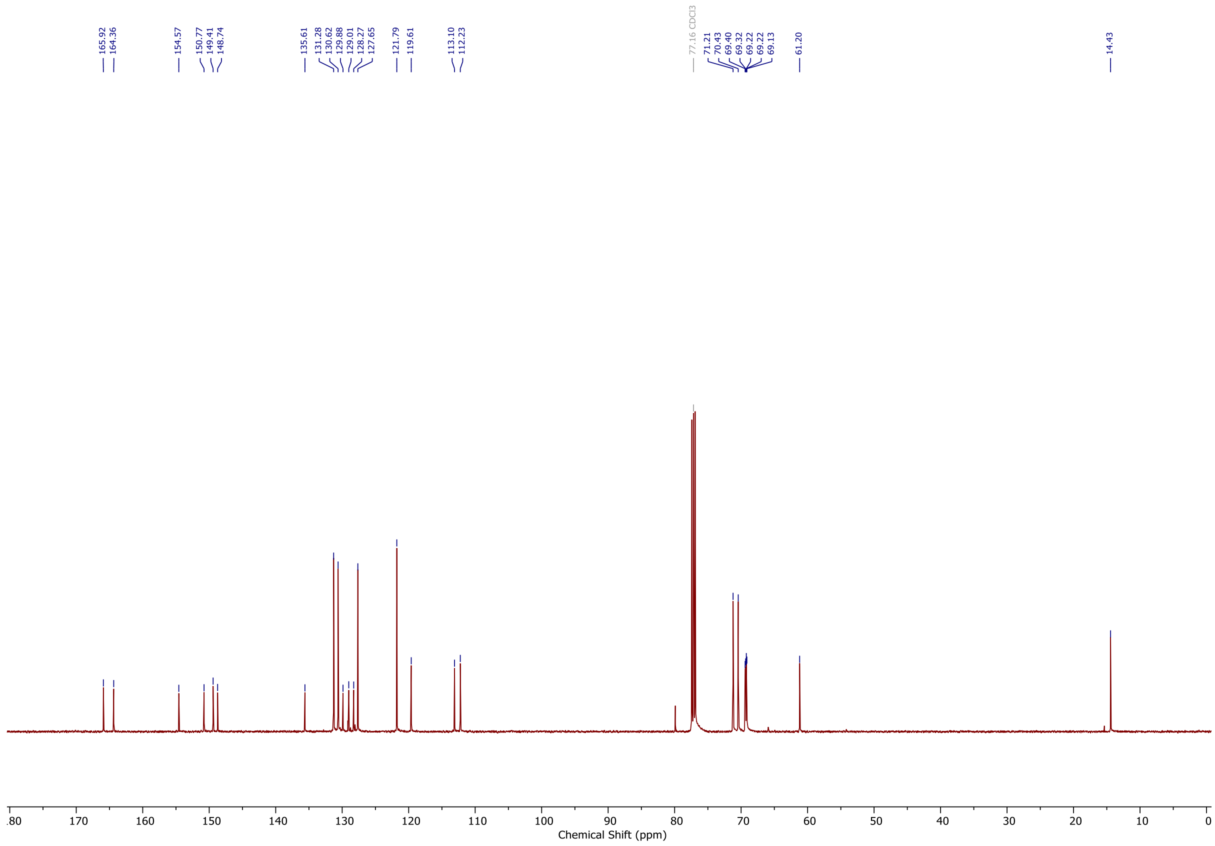

Figure S8. ^{13}C NMR Spectrum of **1·XB** ($CDCl_3$, 126 MHz, 298K).

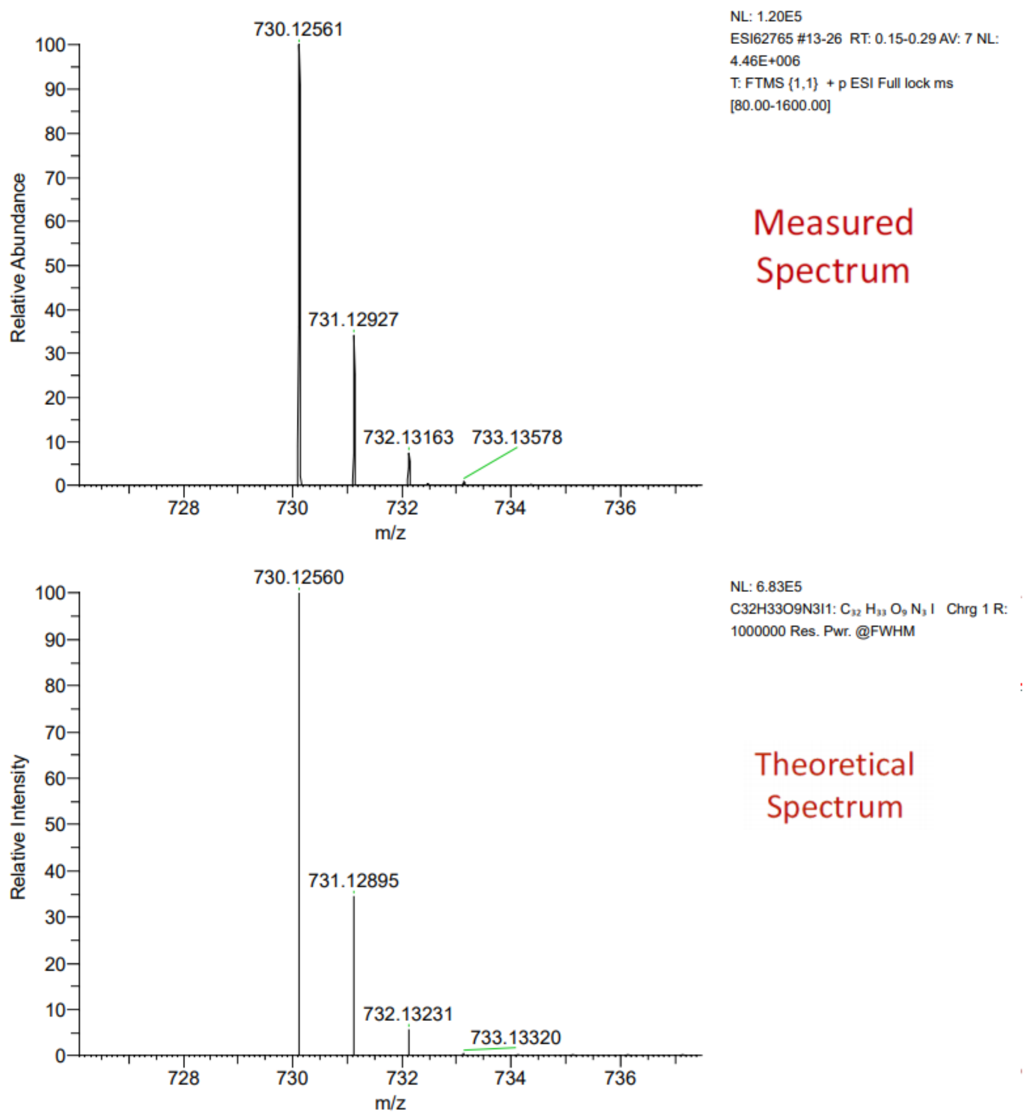

Figure S9. HRESI spectrum of **1·XB**.

1·HB

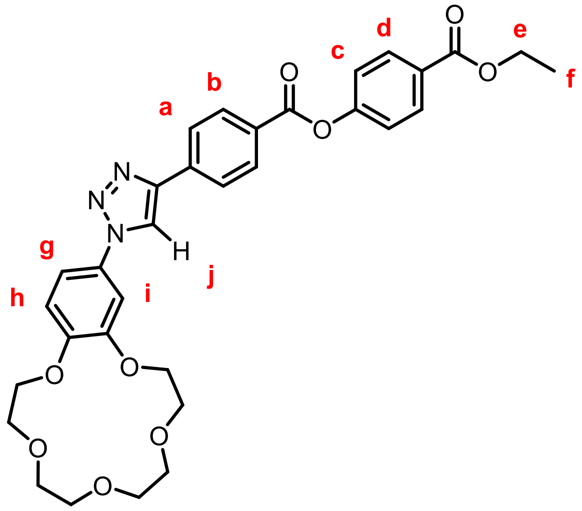

^{1}H NMR (400 MHz, $CDCl_3$) δ 8.28 (s, $1H_j$), 8.25 (m, $2H_b$), 8.16 – 8.11 (m, $2H_d$), 8.08 – 8.02 (m, $2H_a$), 7.38 (m, $1H_i$), 7.36 – 7.28 (m, $2H_c$), 7.22 (dd, *J* = 8.6, 2.5 Hz, $1H_g$), 6.97 (d, *J* = 8.6 Hz, $1H_h$), 4.39 (q, *J* = 7.1 Hz, $2H_e$), 4.21 (m, 4H), 3.94 (m, 4H), 3.87 – 3.71 (m, 16H), 1.40 (t, *J* = 7.1 Hz, $3H_f$).

^{13}C NMR (101 MHz, $CDCl_3$) δ 165.97, 164.42, 154.63, 150.09, 149.88, 147.06, 135.72, 131.31, 131.07, 130.75, 128.73, 128.29, 125.90, 121.83, 119.09, 113.98, 113.09, 107.26, 71.24, 70.51 (d, *J* = 3.2 Hz), 69.51, 69.39 (d, *J* = 2.5 Hz), 69.31, 61.23, 14.46.

HRMS (ESI+ve) m/z: 604.22856 ($[M+H]^+$, $C_{32}H_{34}O_9N_3$ requires 604.22896).

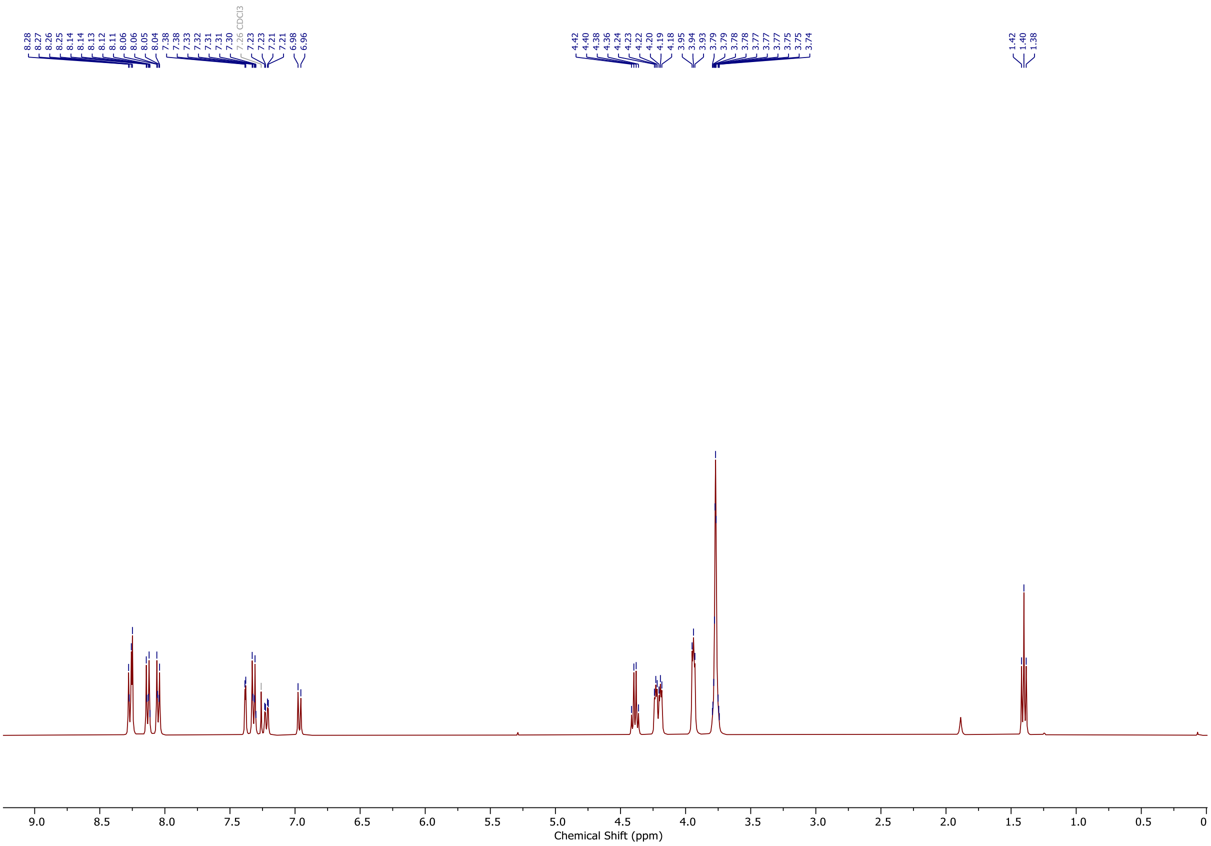

Figure S10. ^{1}H NMR Spectrum of **1·HB** ($CDCl_3$, 400 MHz, 298K).

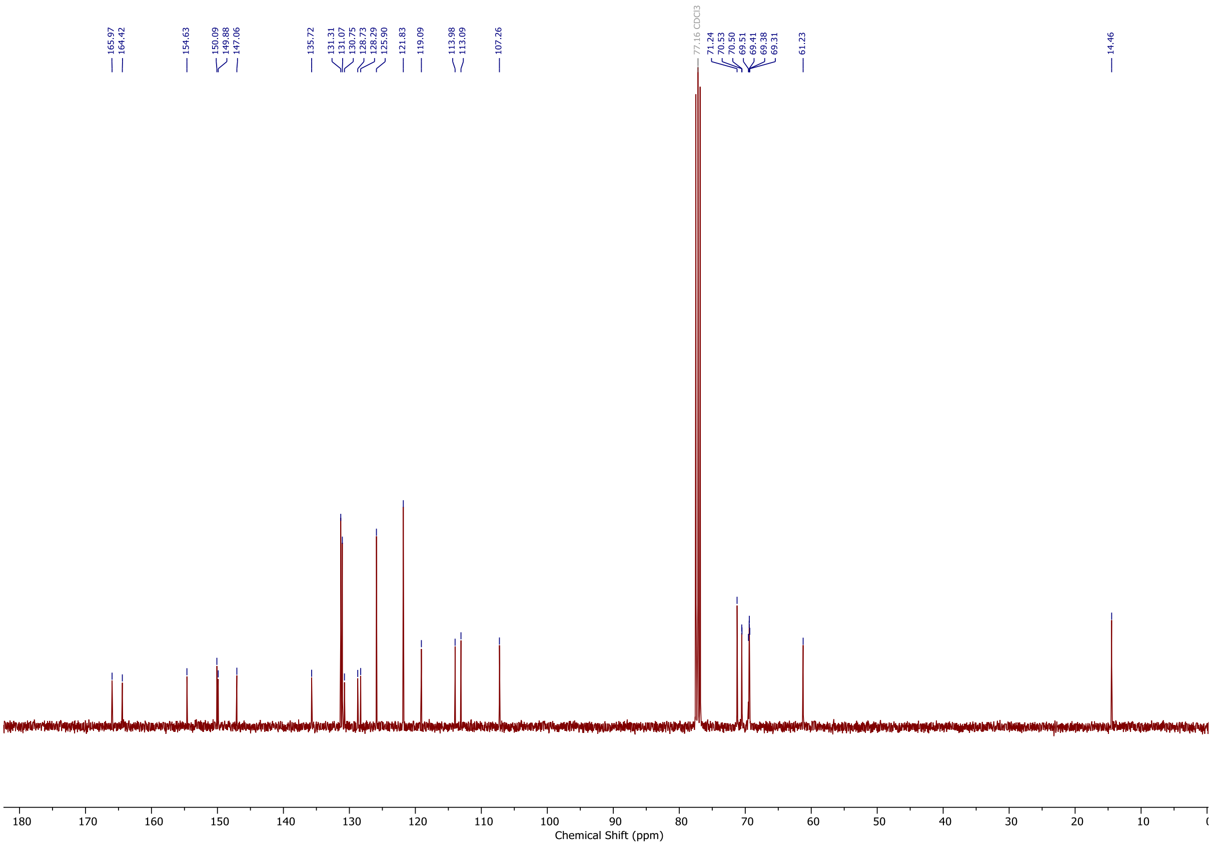

Figure S11. ^{13}C NMR Spectrum of **1·HB** ($CDCl_3$, 101 MHz, 298K).

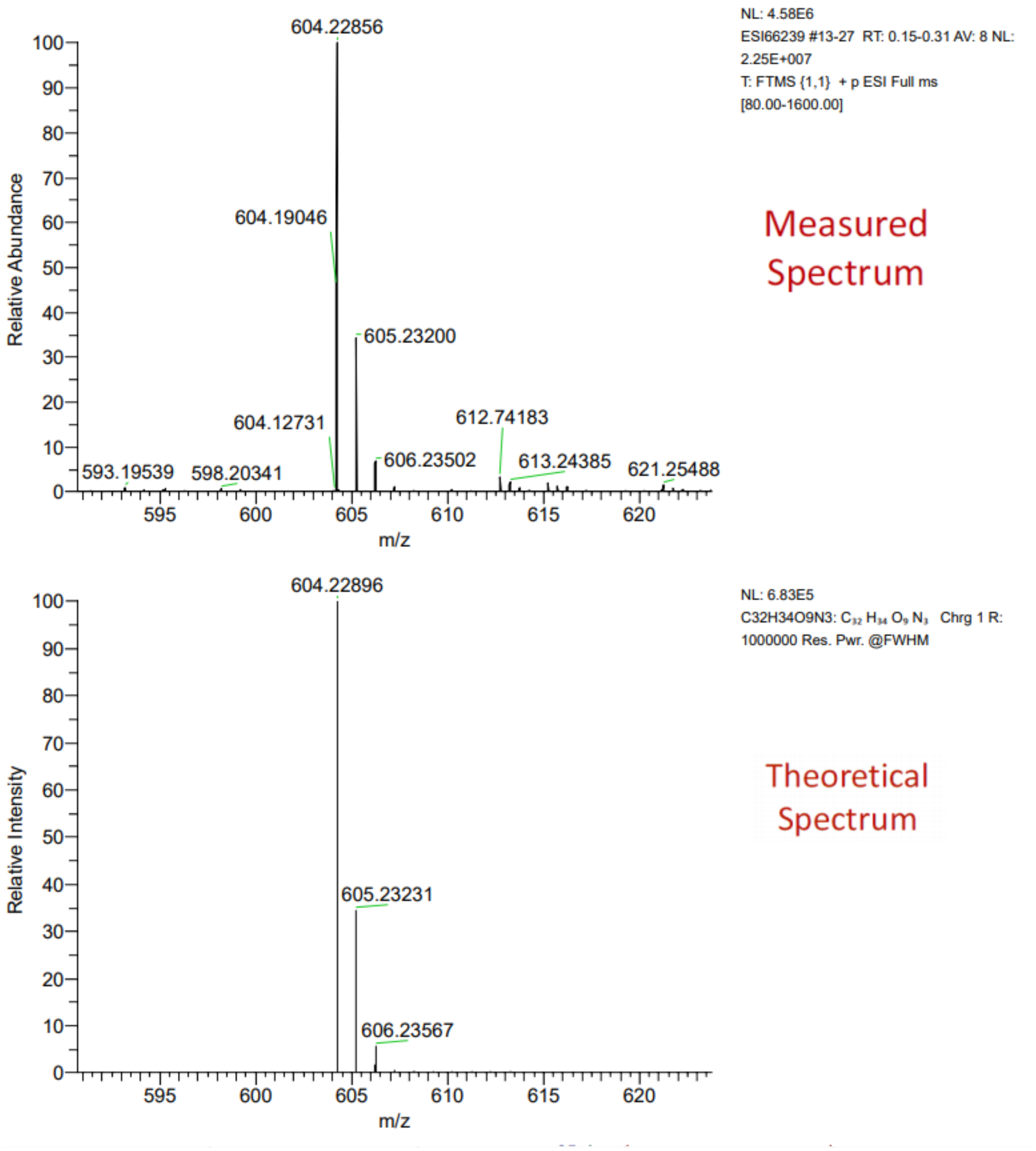

Figure S12. HRESI spectrum of **1·HB**.

9

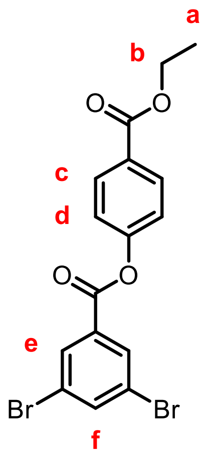

^{1}H NMR (500 MHz, $CDCl_3$) δ 8.26 (d, *J* = 1.8 Hz, $2H_e$), 8.14 (d, *J* = 9.2 Hz, $2H_c$), 7.94 (t, *J* = 1.8 Hz, $1H_f$), 7.29 (d, *J* = 9.2 Hz, $2H_d$), 4.40 (q, *J* = 7.1 Hz, $2H_b$), 1.41 (t, *J* = 7.1 Hz, $3H_a$).

^{13}C NMR (126 MHz, $CDCl_3$) δ 165.84, 162.32, 154.08, 139.30, 132.48, 132.03, 131.45, 128.76, 123.46, 121.60, 61.34, 14.48.

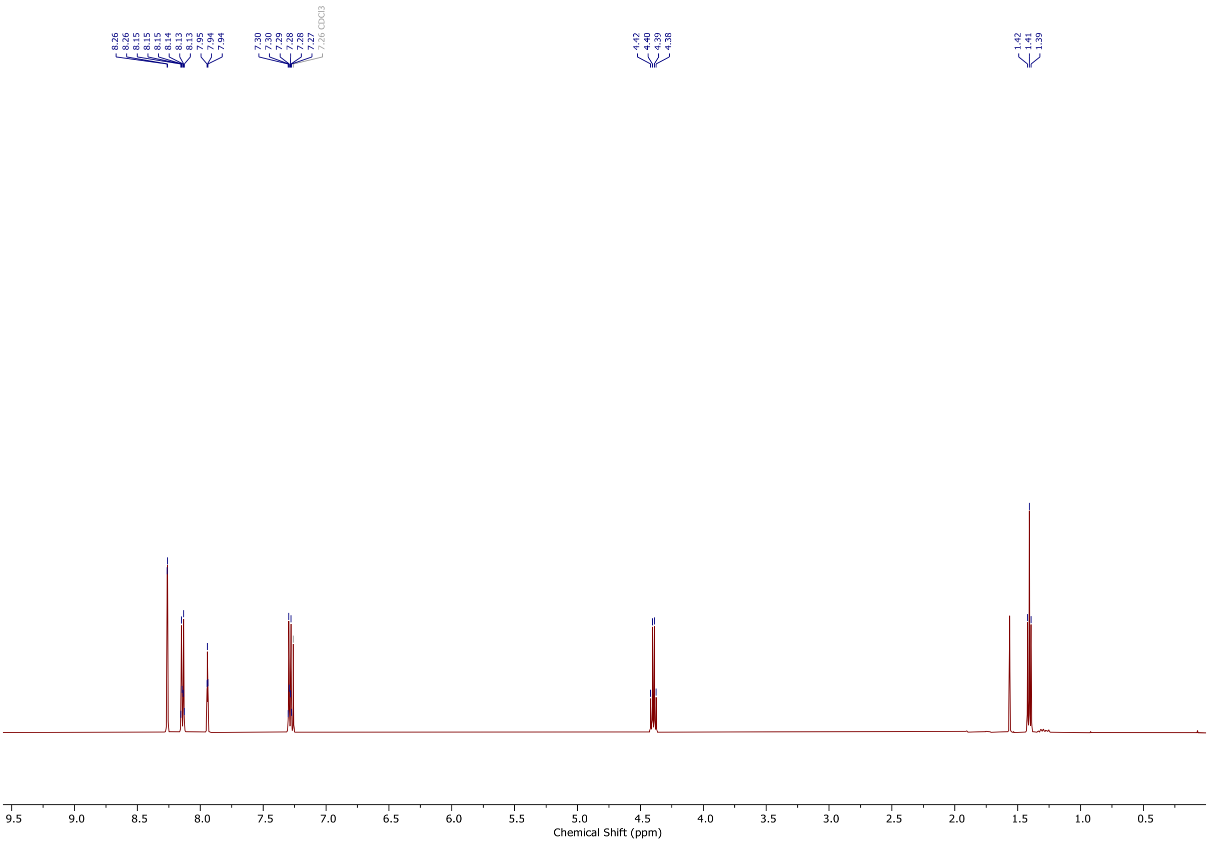

Figure S13. ^{1}H NMR Spectrum of **9** ($CDCl_3$, 500 MHz, 298K).

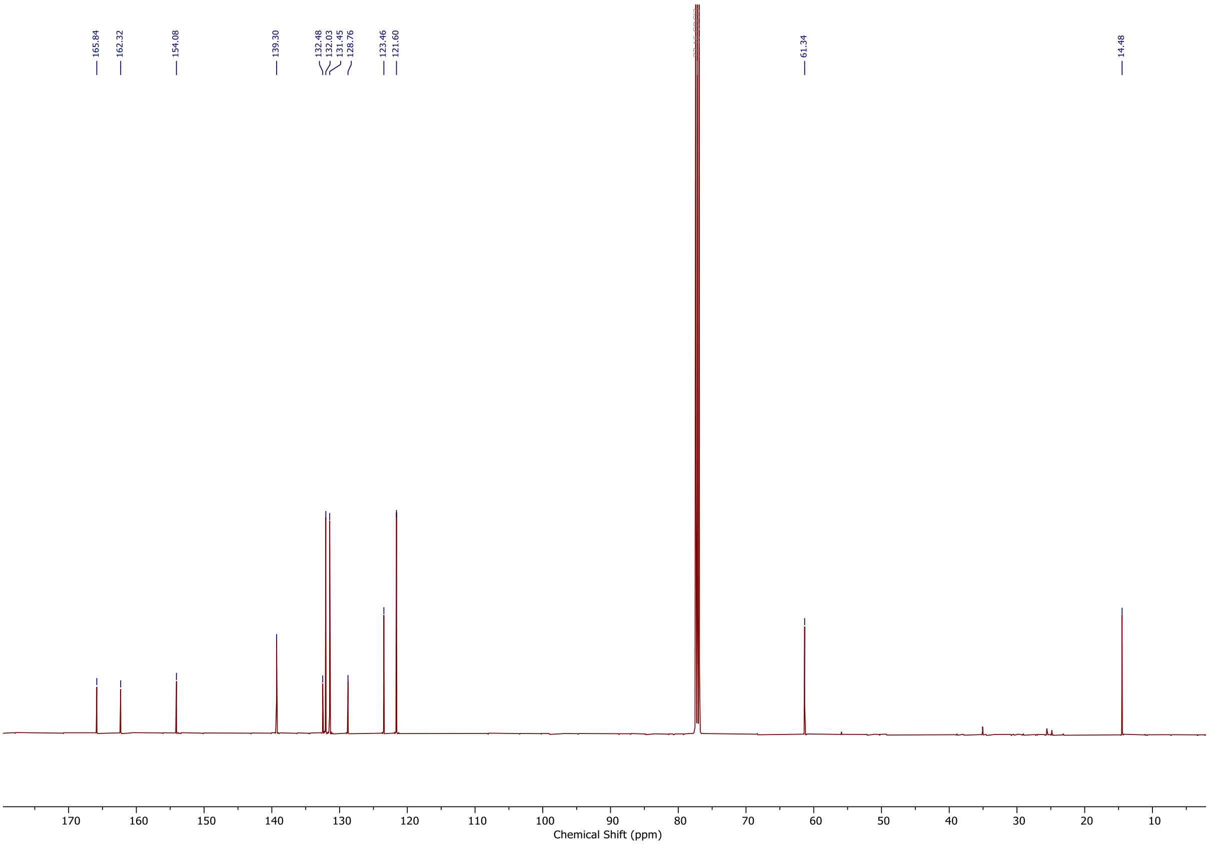

Figure S14. ^{13}C NMR Spectrum of **9** ($CDCl_3$, 126 MHz, 298K).

10

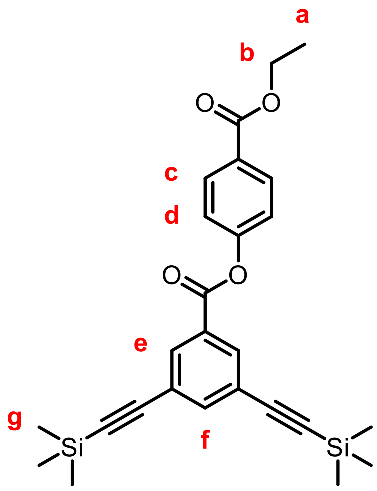

9 (1.00 g, 2.34 mmol), $Pd(PPh_3)_2Cl_2$ (164 mg, 0.234 mmol) and CuI (45 mg, 0.234 mmol) were dissolved in an anhydrous and degassed mixture of THF (20 ml) and NEt_3 (5 ml). The resultant mixture was left to stir at room temperature overnight under an atmosphere of N_2. After which time the crude mixture was filtered through a celite plug and diluted with CH_2Cl_2 (150 ml), the filtrate was washed with 0.1M EDTA/$NH_4OH_{(aq)}$ (25 ml) and H_2O (25 ml). The organic phase was dried over $MgSO_4$ concentrated to dryness and purified by silica gel column chromatography to afford **10** as an off white solid (768 mg, 1.66 mmol, 71%).

^{1}H NMR (400 MHz, $CDCl_3$) δ 8.19 (d, *J* = 1.6 Hz, 2H_f), 8.13 (d, *J* = 8.8 Hz, 2H_c), 7.81 (t, *J* = 1.6 Hz, 1H)$_f$, 7.29 (d, *J* = 8.8 Hz, 1H_d), 4.40 (q, *J* = 7.1 Hz, 2H_b), 1.41 (t, *J* = 7.1 Hz, 3H_a), 0.26 (s, 18H_g).

^{13}C NMR (126 MHz, $CDCl_3$) δ 165.94, 163.45, 154.35, 140.03, 133.24, 131.40, 129.75, 128.52, 124.43, 121.72, 102.75, 96.89, 61.29, 14.49, -0.06.

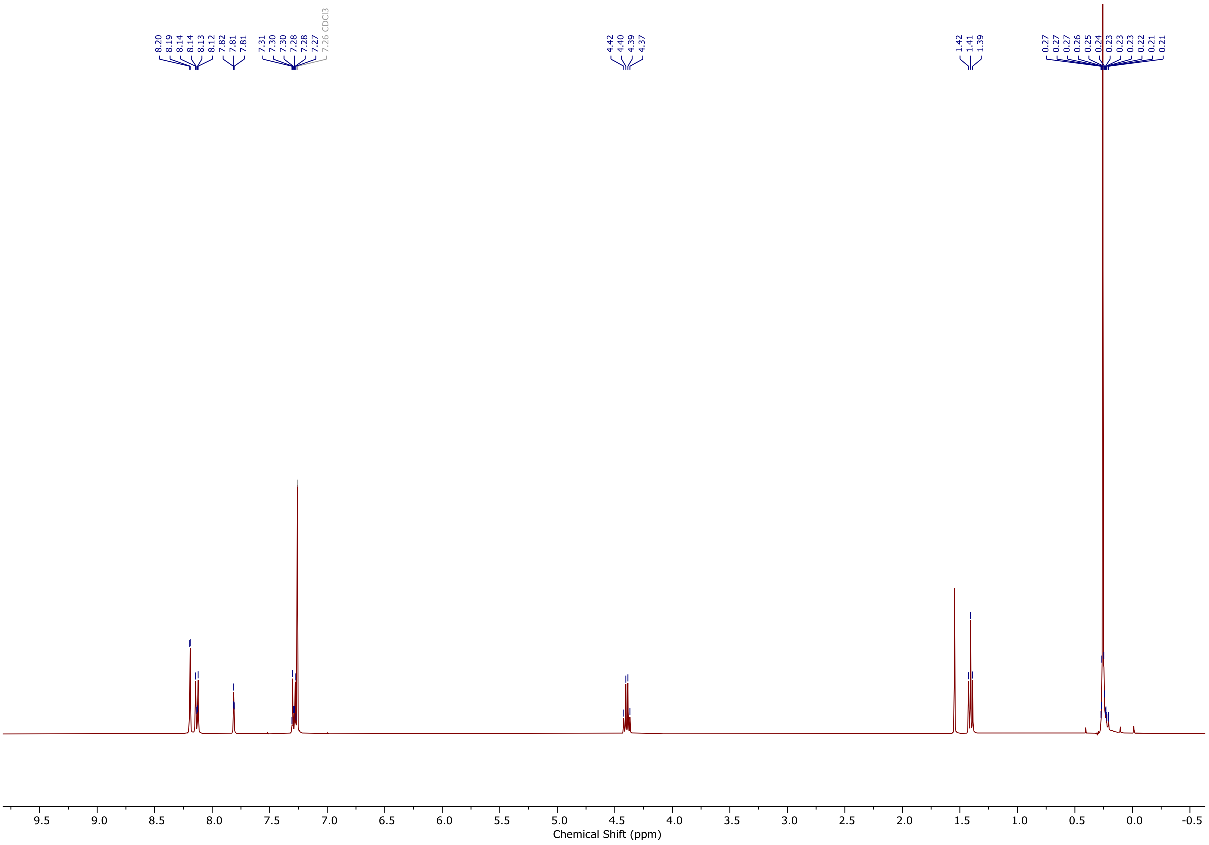

Figure S15. ^{1}H NMR Spectrum of **10** ($CDCl_3$, 400 MHz, 298K).

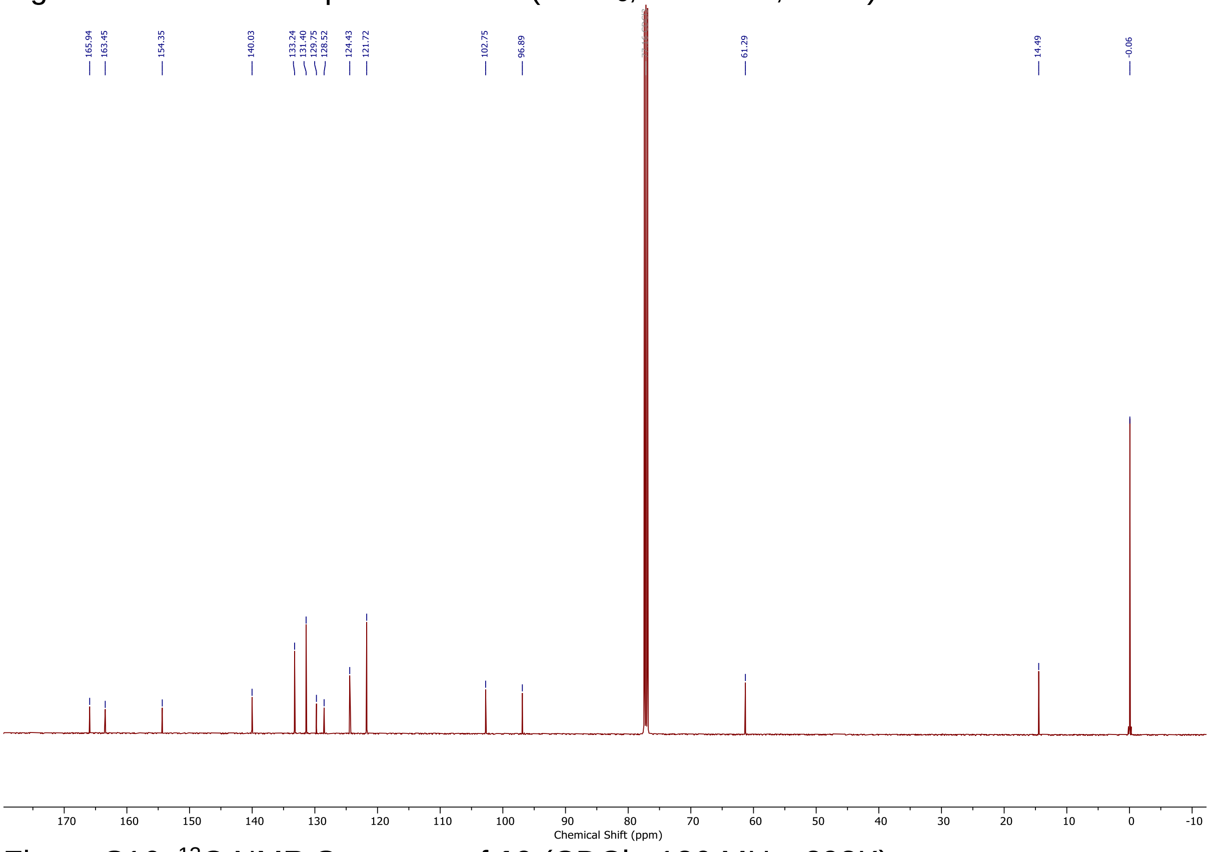

Figure S16. ^{13}C NMR Spectrum of **10** ($CDCl_3$, 126 MHz, 298K).

4

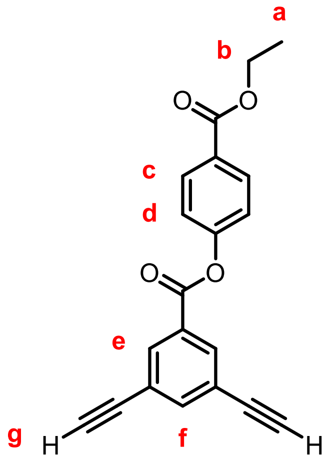

^{1}H NMR (400 MHz, $CDCl_3$) δ 8.27 (d, *J* = 1.6 Hz, $2H_e$), 8.14 (d, *J* = 8.6 Hz, $2H_c$), 7.85 (t, *J* = 1.6 Hz, $1H_f$), 7.30 (d, *J* = 8.6 Hz, $2H_d$), 4.39 (t, *J* = 7.1 Hz, $2H_b$), 3.18 (s, $2H_g$), 1.41 (t, *J* = 7.1 Hz, $3H_a$).

^{13}C NMR (101 MHz, $CDCl_3$) δ 165.89, 163.21, 154.26, 140.25, 133.86, 131.41, 130.07, 128.60, 123.57, 121.67, 81.46, 79.54, 61.30, 14.48.

HRMS (ESI+ve) m/z: 319.09661 ($[M+H]^+$, $C_{20}H_{15}O_4$ requires 319.09649).

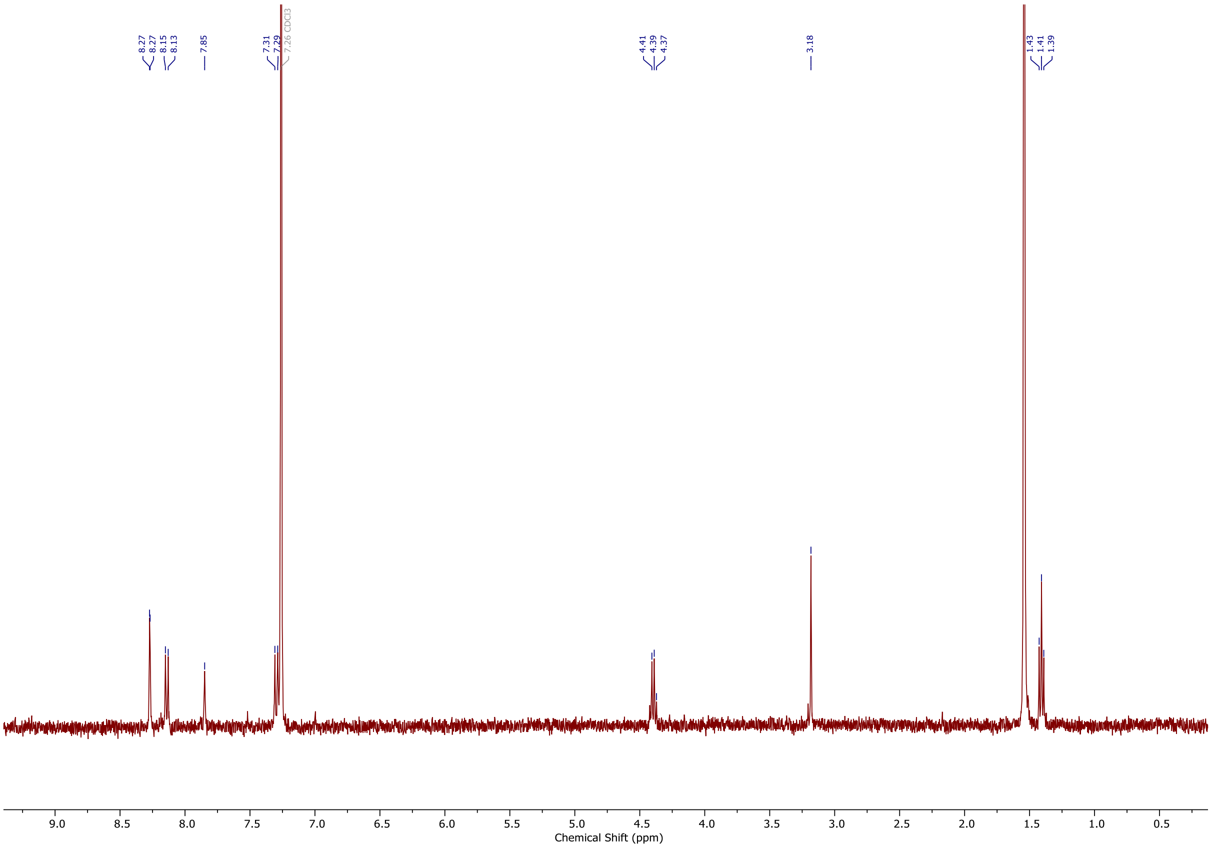

Figure S17. ^{1}H NMR Spectrum of **4** ($CDCl_3$, 400 MHz, 298K).

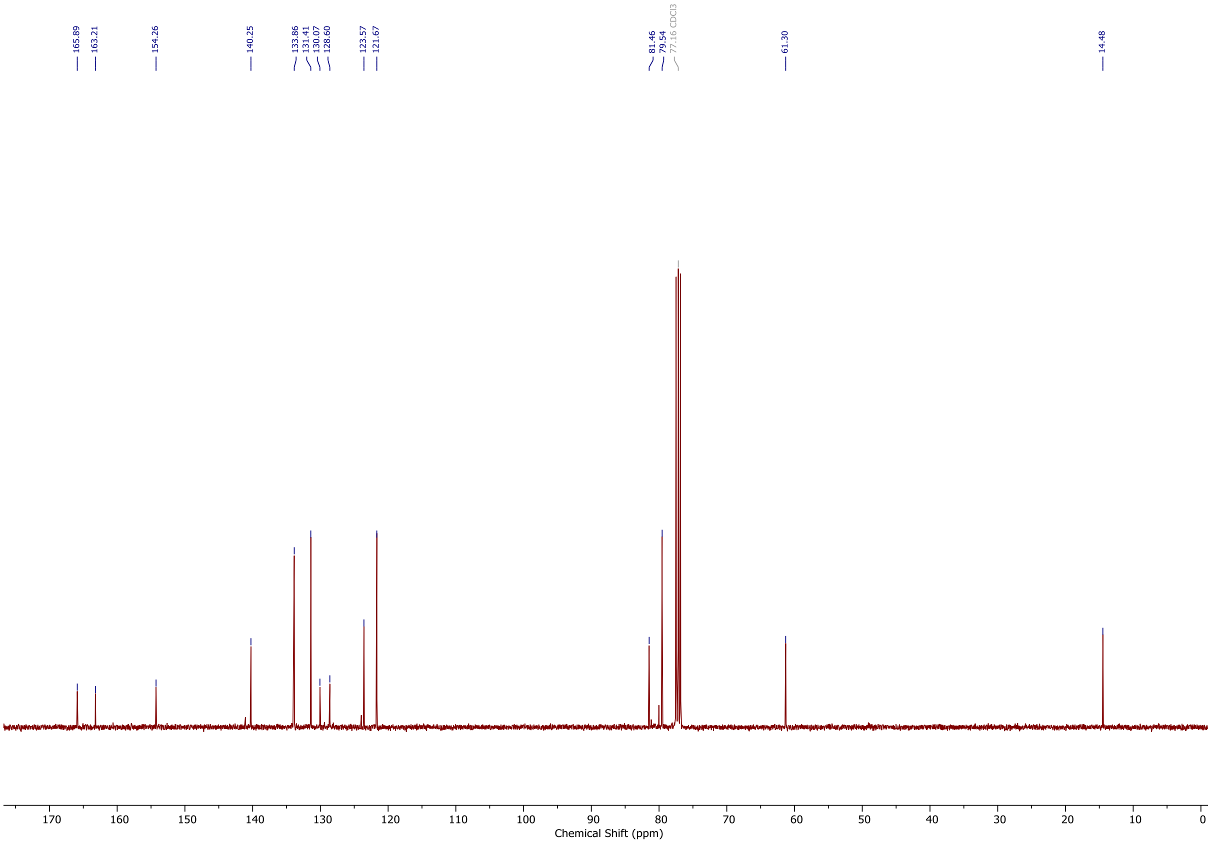

Figure S18. ^{13}C NMR Spectrum of **4** ($CDCl_3$, 101 MHz, 298K).

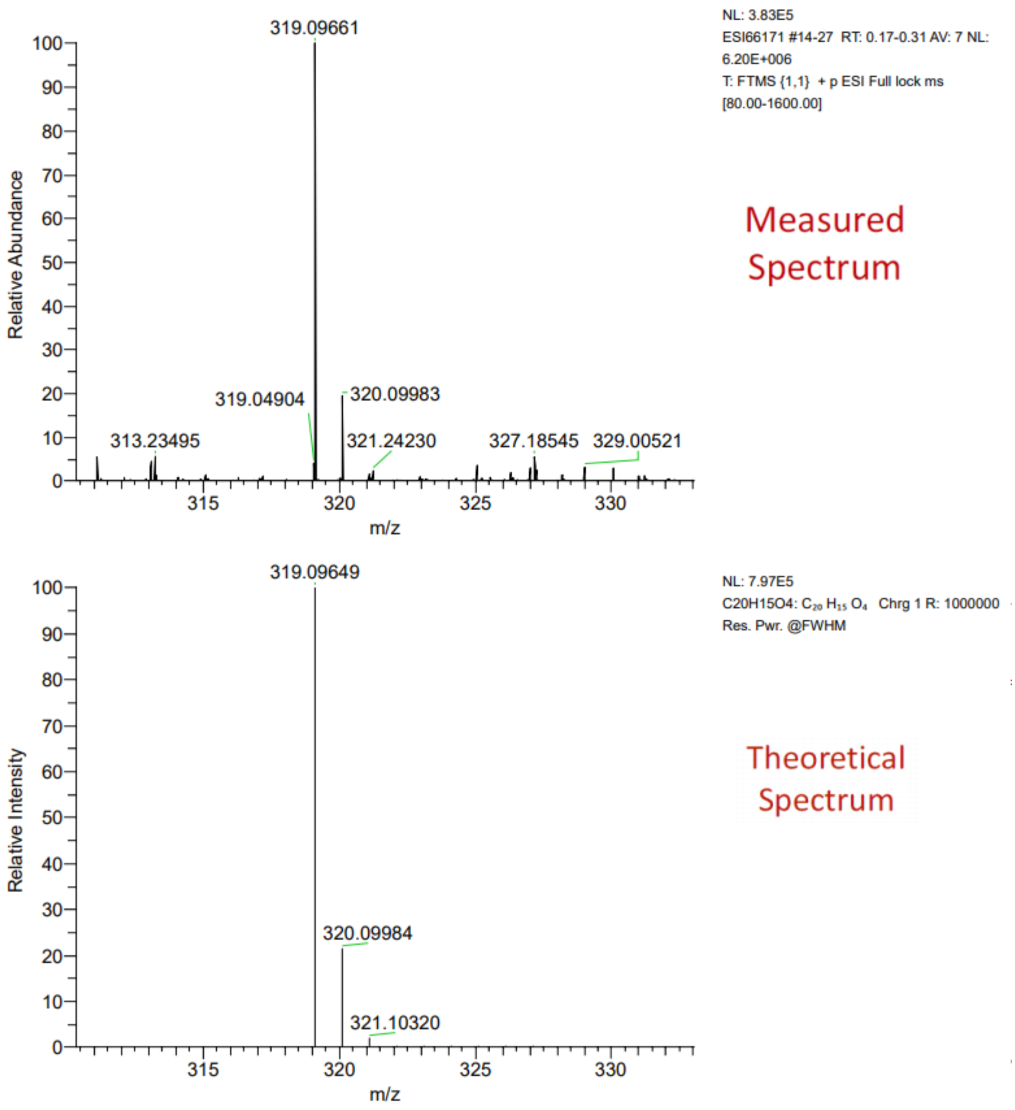

Figure S19. HRESI spectrum of **4**.

11

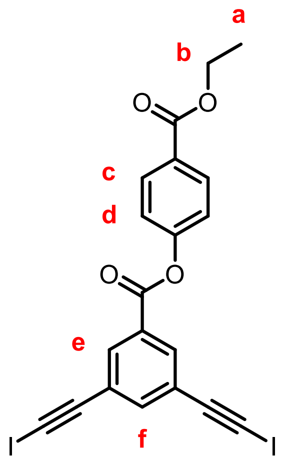

10 (500 mg, 1.06 mmol) was dissolved in anhydrous DMF (15 ml), to which was added $AgNO_3$ (36 mg, 0.212 mmol) and NIS (716 mg, 3.18 mmol), the mixture was left to stir for 4 hours at room temperature excluded from light. After which time the reaction mixture was diluted with H_2O (500 ml) and the resultant precipitate isolated by filtration under vacuum. The obtained solid was copiously washed with water, the solid was then dissolved in CH_2Cl_2 dried over $MgSO_4$ and solvent removed in vacuo to afford **11** as yellow solid (508 mg, 0.890 mmol, 84%).

^{1}H NMR (500 MHz, $CDCl_3$) δ 8.18 (d, *J* = 1.6 Hz, $2H_e$), 8.16 – 8.10 (m, $2H_c$), 7.73 (t, *J* = 1.6 Hz, $1H_f$), 7.31 – 7.27 (m, $2H_d$), 4.39 (q, *J* = 7.1 Hz, $2H_b$), 1.41 (t, *J* = 7.1 Hz, $3H_a$).

^{13}C NMR (126 MHz, $CDCl_3$) δ 165.90, 163.16, 154.24, 140.59, 134.00, 131.41, 129.90, 128.58, 124.65, 121.65, 92.03, 61.31, 14.48, 10.31.

HRMS (ESI+ve) m/z: 570.88970 ($[M+H]^+$, $C_{20}H_{13}O_4I_2$ requires 570.88977).

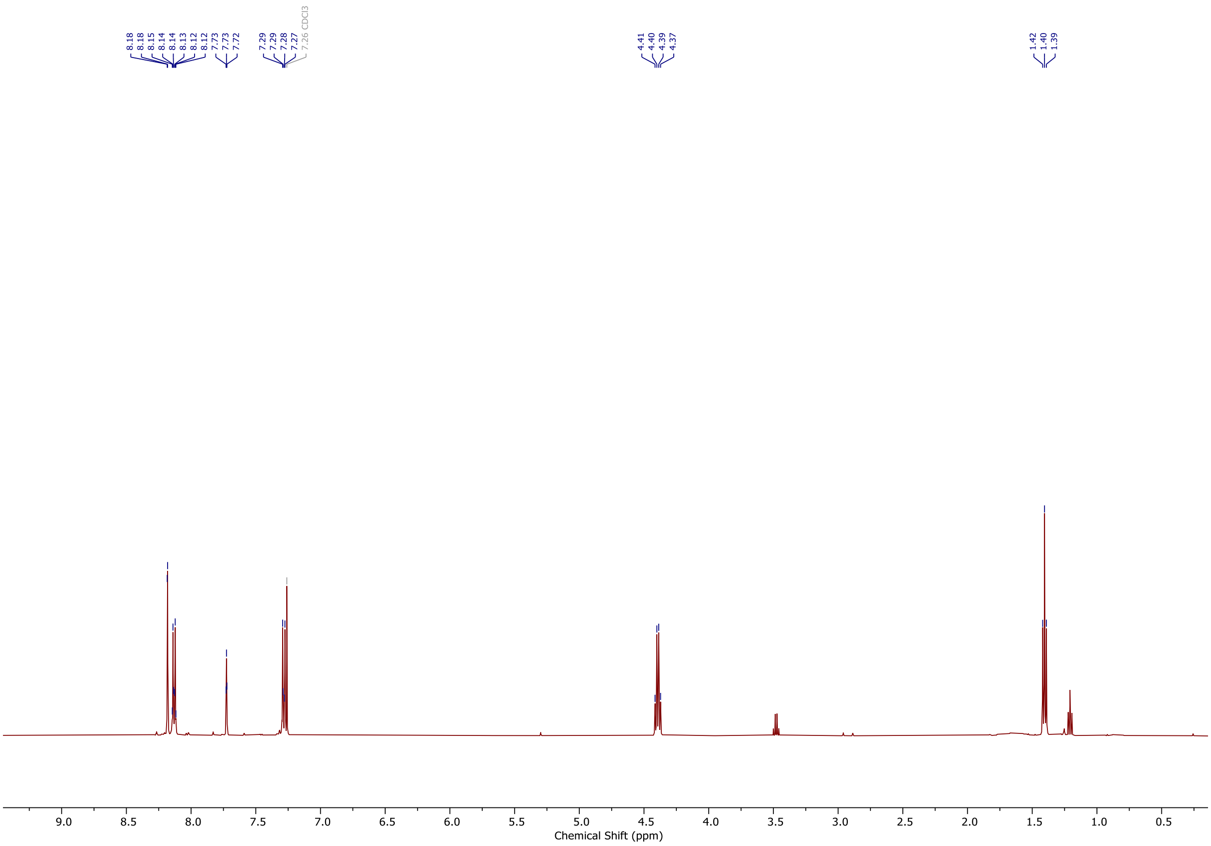

Figure S20. ^{1}H NMR Spectrum of **11** ($CDCl_3$, 500 MHz, 298K).

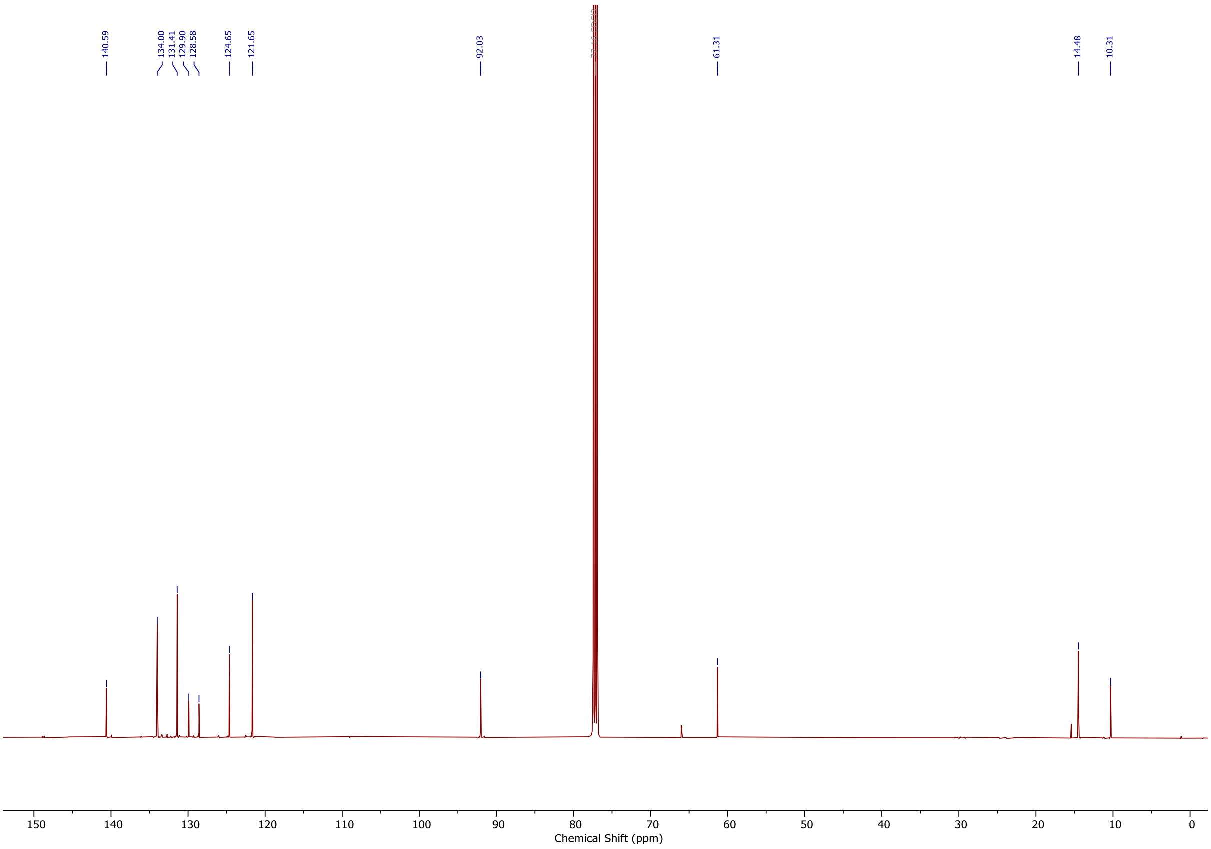

Figure S21. ^{13}C NMR Spectrum of **11** ($CDCl_3$, 126 MHz, 298K).

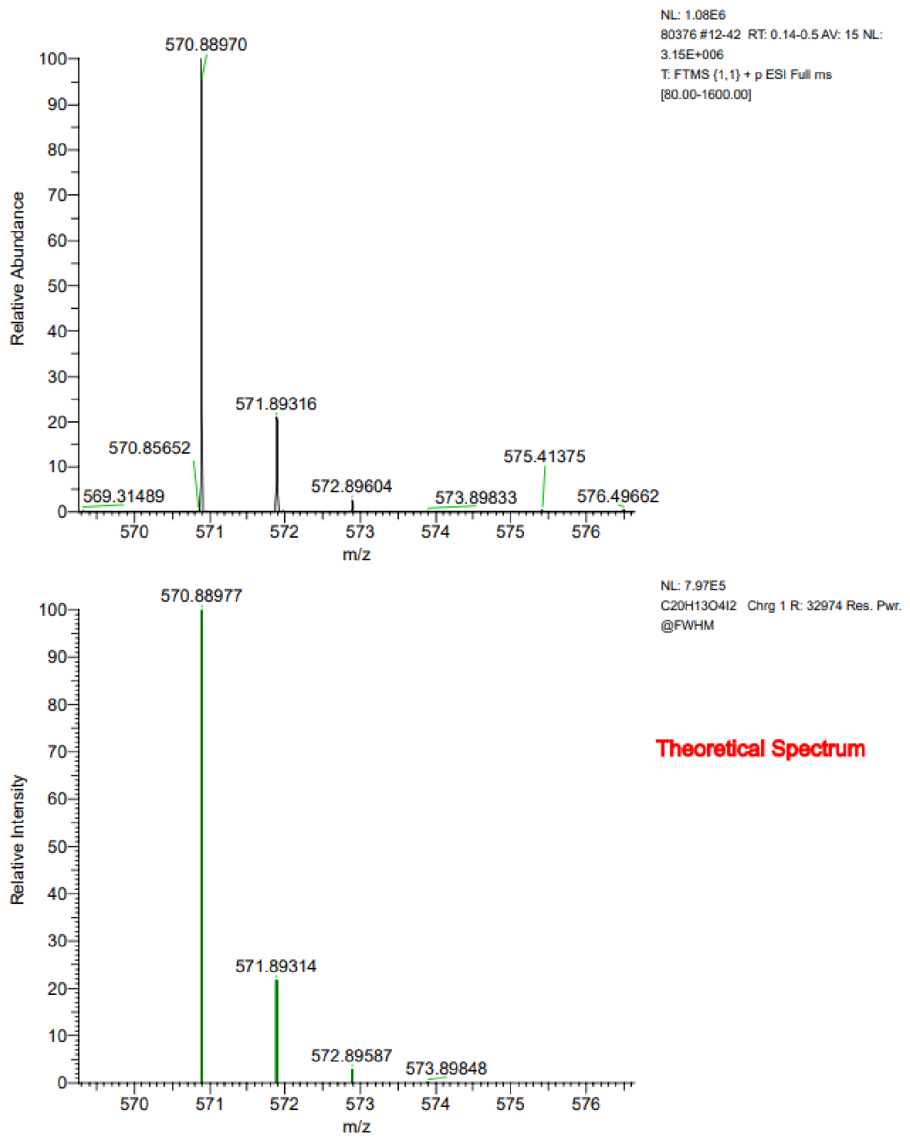

Figure S22. HRESI spectrum of **11**.

2·HB

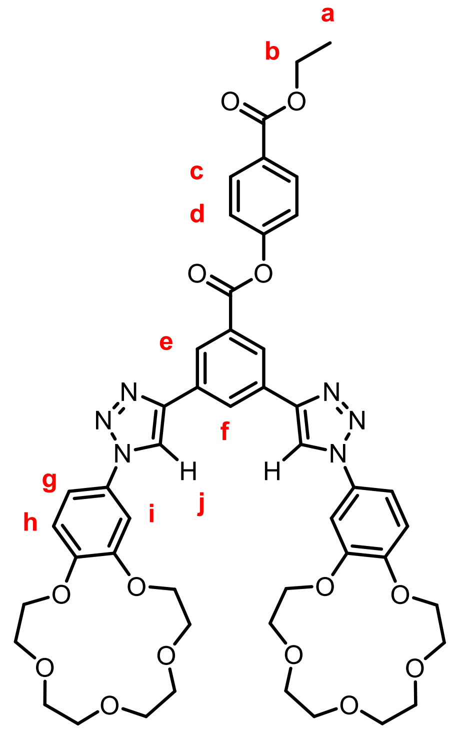

^{1}H NMR (500 MHz, DMSO) δ 9.06 (s, 2H_j), 8.45 (d, J = 1.8 Hz, 1H_f), 8.18 (d, J = 1.6 Hz, 2H_e), 7.64 (d, J = 8.7 Hz, 2H_c), 7.15 – 7.04 (m, 6$H_{g,h,i}$), 6.71 (d, J = 8.7 Hz, 2H_d), 3.90 (q, J = 7.1 Hz, 2H_b), 3.75 (s, 4H), 3.68 (s, 4H), 3.36 (s, 8H), 3.26 – 3.14 (m, 16H), 0.90 (t, J = 7.1 Hz, 3H_a).

^{13}C NMR (126 MHz, DMSO) δ 165.07, 163.85, 154.32, 149.09, 148.75, 145.70, 132.02, 130.88, 130.37, 130.08, 127.83, 125.84, 122.55, 120.58, 113.83, 112.36, 106.02, 70.53, 69.70 (d, J = 2.7 Hz), 68.79, 68.71 (d, J = 2.9 Hz), 68.65, 60.94, 14.18.

HRMS (ESI+ve) m/z: 937.36159 ($[M+H]^+$, $C_{48}H_{53}O_{15}N_6$ requires 937.36143).

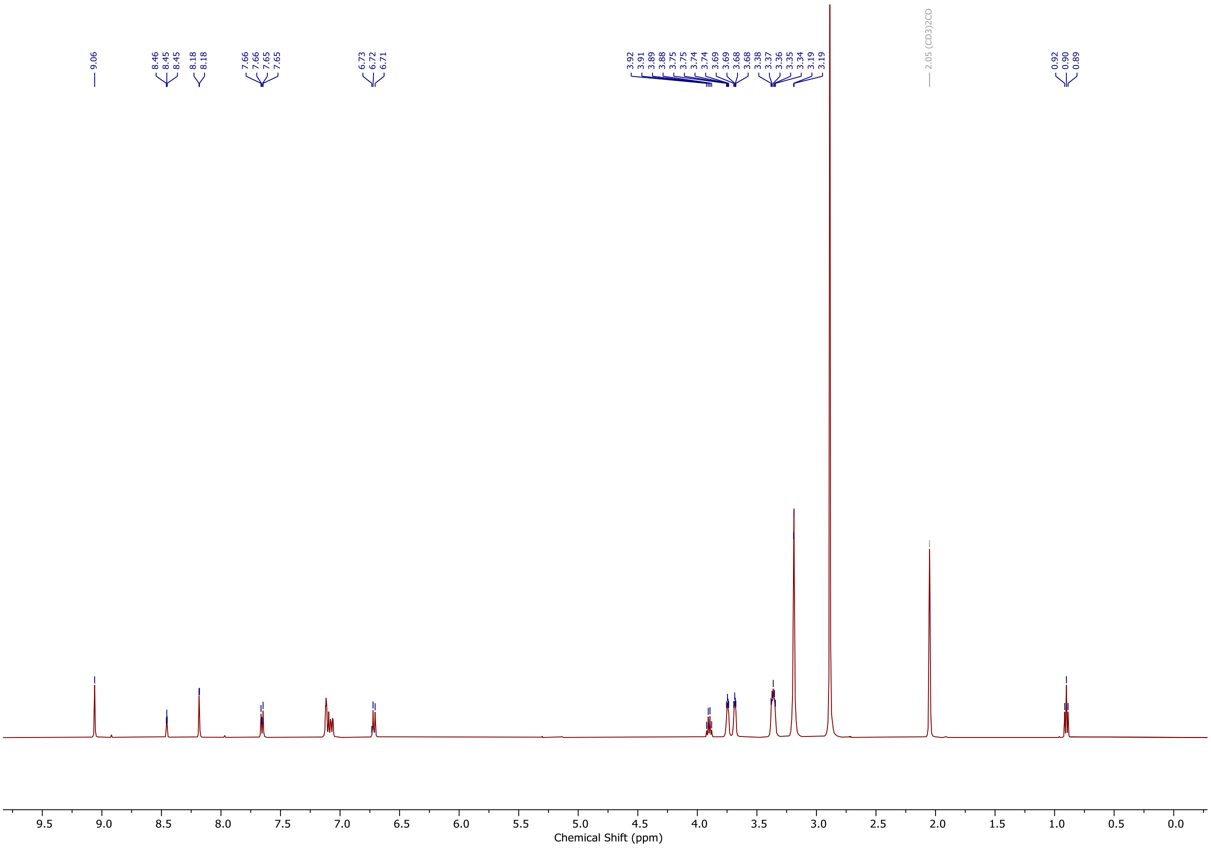

Figure S23. ^{1}H NMR Spectrum of **2·HB** ($CDCl_3$, 500 MHz, 298K).

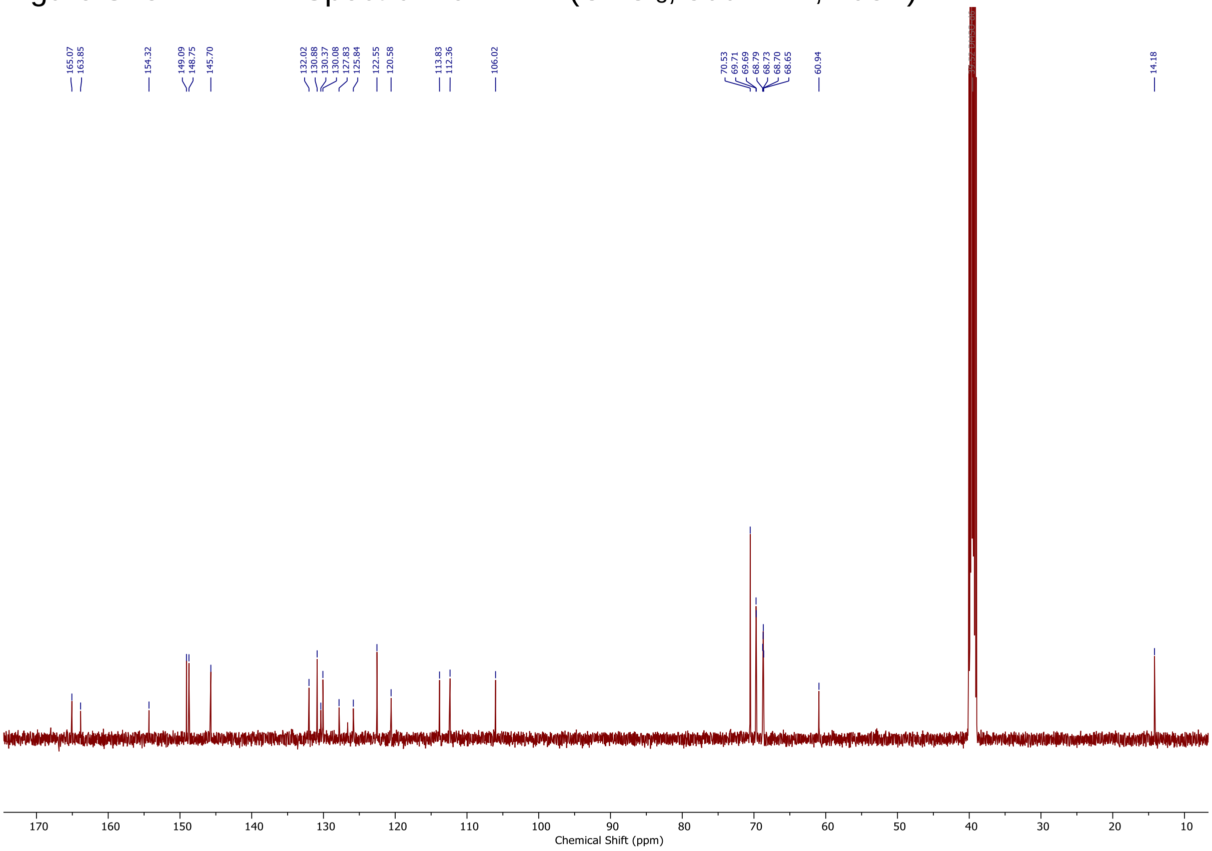

Figure S24. ^{13}C NMR Spectrum of **2·HB** ($CDCl_3$, 126 MHz, 298K).

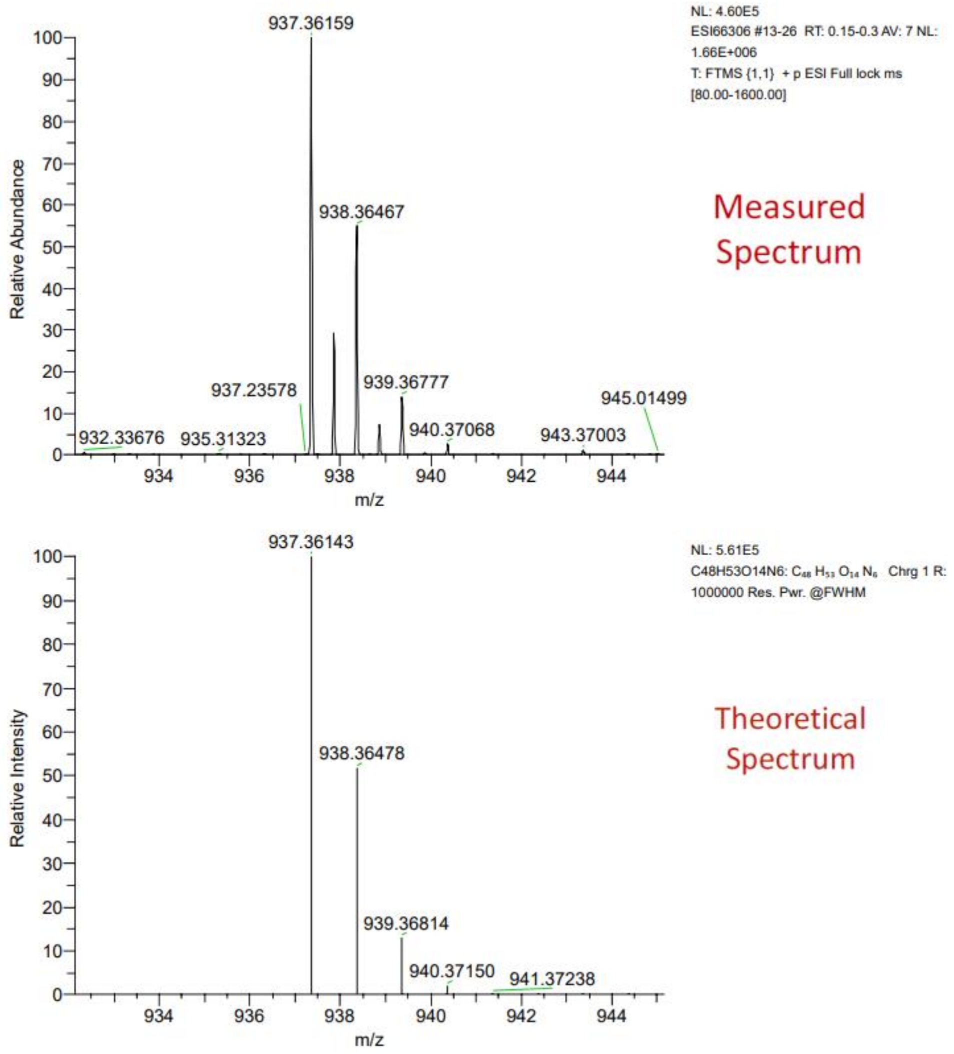

Figure S25. HRESI spectrum of **2·HB**.

2·XB

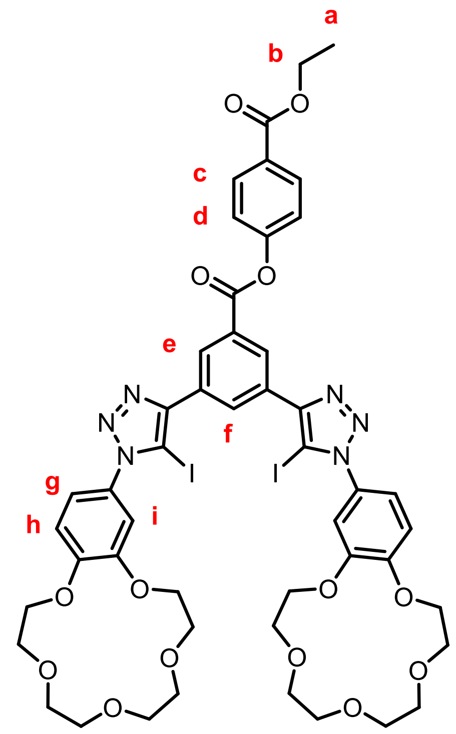

^{1}H NMR (400 MHz, $CDCl_3$) δ 8.98 (s, $1H_f$), 8.92 (s, $2H_e$), 8.14 (d, J = 8.4 Hz, $2H_c$), 7.36 (d, J = 8.4 Hz, $2H_d$), 7.16 – 6.91 (m, $6H_{g,h,i}$), 4.39 (q, J = 7.1 Hz, $2H_b$), 4.28 – 4.08 (m, 8H), 3.94 (m, 8H), 3.77 (m, 16H), 1.40 (t, J = 7.1 Hz, $3H_a$).

^{13}C NMR (101 MHz, $CDCl_3$) δ 165.97, 164.13, 154.56, 150.71, 149.38, 148.76, 131.60, 131.33, 130.27, 129.98, 129.34, 128.36, 121.82, 119.61, 113.08, 112.18, 71.16, 70.42, 69.58 – 68.99 (m), 61.24, 14.46.

HRMS (ESI+ve) m/z: 937.36159 ($[M+H]^+$, $C_{48}H_{53}O_{15}N_6$ requires 937.36143).

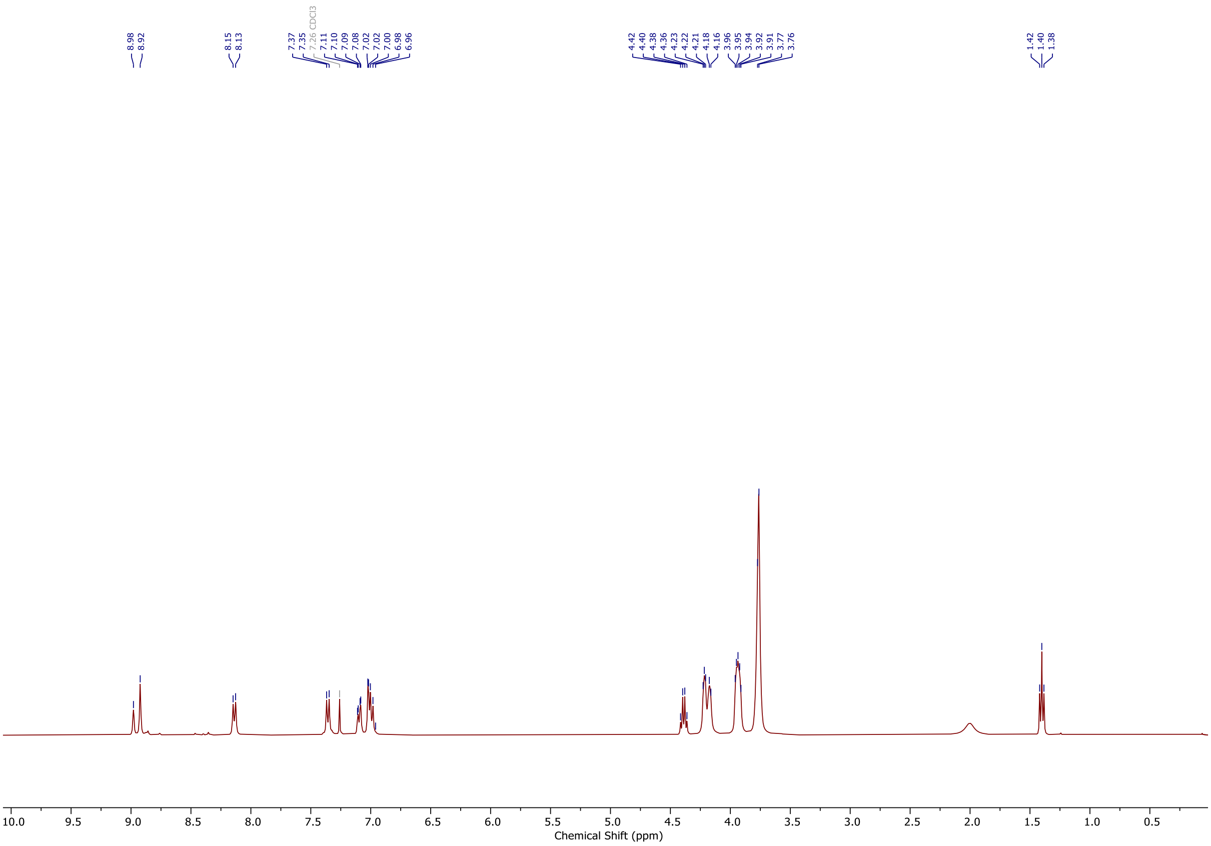

Figure S26. ^{1}H NMR Spectrum of **2·XB** ($CDCl_3$, 400 MHz, 298K).

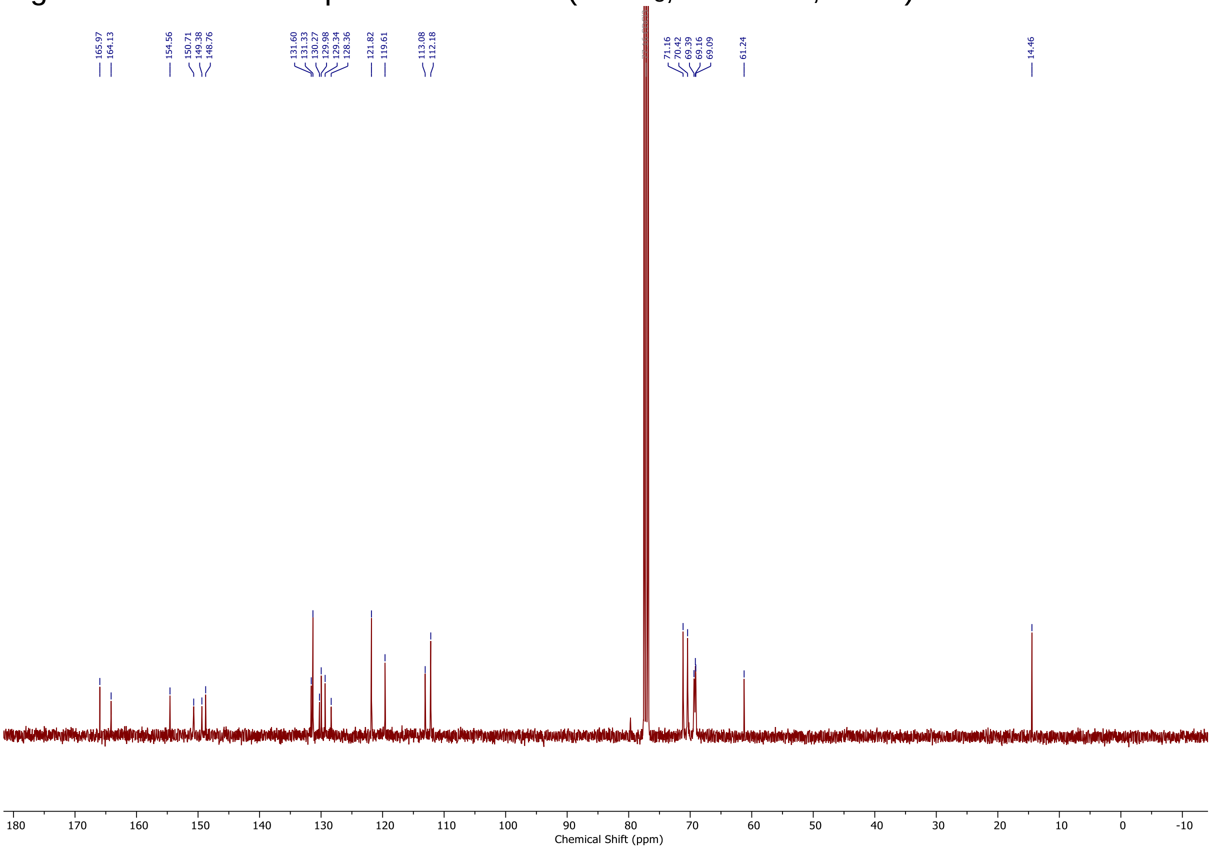

Figure S27. ^{13}C NMR Spectrum of **2·XB** ($CDCl_3$, 126 MHz, 298K).

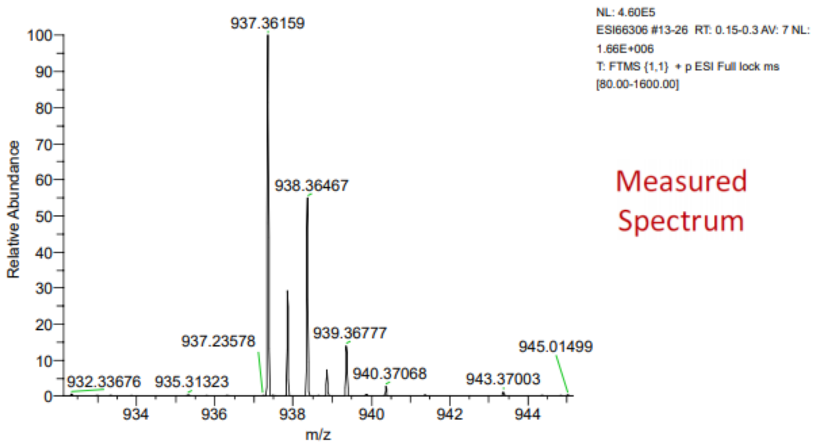

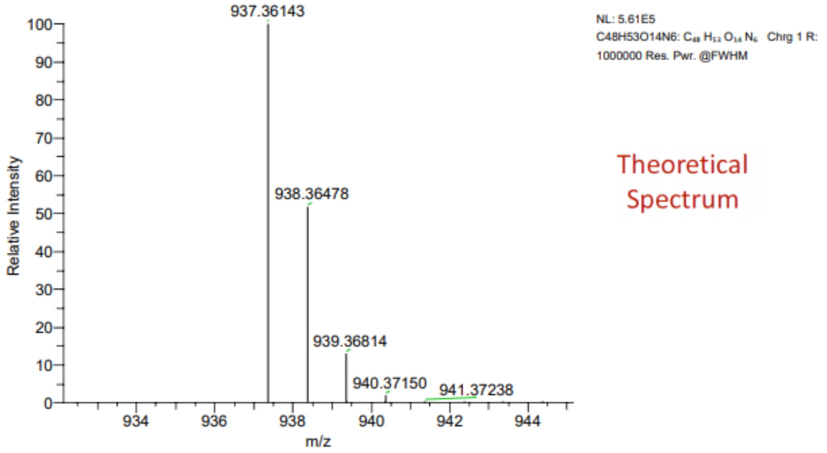

Figure S28. HRESI spectrum of **2·XB**.

References

[1] A. T. Wright, J. D. Song, B. F. Cravatt, *J. Am. Chem. Soc.* **2009**, *131*, 10692–10700.

[2] Y. Jin, D. Watkins, N. N. Degtyareva, K. D. Green, M. N. Spano, S. Garneau-Tsodikova, D. P. Arya, *MedChemComm* **2016**, *7*, 164–169.

[3] A. J. Pearson, J. B. Kim, *Tetrahedron Lett.* **2003**, *44*, 8525–8527.

[4] T. Bunchuay, A. Docker, U. Eiamprasert, P. Surawatanawong, A. Brown, P. D. Beer, *Angew. Chem. Int. Ed.* **2020**, *59*, 12007–12012.
